# Goal Location Beta Oscillations and Sharp Wave–Ripples Are Complementary Markers of Learning-Related Changes in Hippocampal–Neocortical Networks

**DOI:** 10.1523/ENEURO.0048-26.2026

**Published:** 2026-09-29

**Authors:** Sameera Shridhar, Zachary M. Leveroni, Nan Zhou, Ellie Quattrocchi, Audrey E. Kaye, Jaquelin Gutierrez, Amanda Rodríguez León, Leslie M. Kay, Jai Y. Yu

**Affiliations:** ^1^Department of Psychology, University of Chicago, Chicago, Illinois 60637; ^2^Institute for Mind and Biology, University of Chicago, Chicago, Illinois 60637; ^3^Neuroscience Institute, University of Chicago, Chicago, Illinois 60637

**Keywords:** beta oscillations, hippocampus, prefrontal cortex, sharp wave–ripples, spatial learning

## Abstract

The hippocampus and neocortex are thought to undergo reorganization to incorporate new information during learning. How can we identify network states that indicate learning is occurring? In the hippocampus, one network signature with these properties is the sharp wave–ripple (SWR), which occurs more frequently during initial learning. However, it remains unclear which neural signatures track changes in neocortical networks during learning. We therefore recorded local field potentials simultaneously from the dorsal hippocampus (CA1) and prefrontal cortex (PFC) as male rats learned spatial navigation tasks. After animals reached goal locations, we found beta frequency (∼20 Hz) oscillations in PFC that increased in power with improvements in performance across days of training. In contrast, the rate and size of hippocampal SWRs decreased over the same period. Furthermore, goal-entry beta in PFC but not in CA1 was correlated with reward likelihood. Our results suggest that neocortical beta oscillations develop gradually as learning occurs, which stands in contrast to the reduction in hippocampal SWRs as the task becomes familiar. We hypothesize that PFC beta oscillations and hippocampal SWRs are complementary markers of experience-dependent change in hippocampal–neocortical networks during learning.

## Significance Statement

Hippocampal and neocortical circuits are crucial for learning. The network states associated with the learning process remain challenging to identify. It remains unclear how to distinguish states associated with active reorganization during learning versus states of stability after learning has taken place. We report that properties of neocortical beta oscillations and hippocampal sharp wave–ripples show an inverse relationship across training. Our results support the hypothesis that these network signatures are complementary learning- and experience-related markers in hippocampal–neocortical networks.

## Introduction

Learning involves incorporating new information from experience into existing knowledge structures. This process is hypothesized to rely on the complementary contributions of the hippocampus and neocortex (McClelland et al., 1995; Kumaran et al., 2016). Hippocampal networks show rapid activity changes under novel conditions to capture new information, whereas neocortical networks are gradually reorganized through coordination with the hippocampus to form enduring memories of new experiences. Whether learning is occurring or has occurred can be assessed by observing changes in performance. At the neural level, however, the network states associated with the learning process remain challenging to identify. How can we determine if these networks are in a state of reorganization associated with learning as opposed to being in a state of stability after learning has taken place?

Network signatures associated with learning can be found in the hippocampus. Goal location hippocampal sharp wave–ripples (SWRs) become more frequent in novel environments (Foster and Wilson, 2006; Cheng and Frank, 2008; Singer and Frank, 2009; Singer et al., 2013; Tang et al., 2017) or when a change occurs in a familiar environment or task (Larkin et al., 2014; Ambrose et al., 2016; Carey et al., 2019; Huelin Gorriz et al., 2023). SWRs are hypothesized to support learning by initiating the reorganization of extrahippocampal networks during awake and sleep states to incorporate new and relevant information (Buzsáki, 1989, 1996, 2015; van der Meer and Bendor, 2025). In contrast, it remains unclear which neocortical network markers can signal learning-related changes during the waking state.

Several lines of evidence point to beta oscillations (15–30 Hz) as a candidate neocortical network marker for learning-related changes. First, in olfactory learning, beta oscillations in the piriform cortex and olfactory bulb (OB) during odor sampling develop with experience in a task (Martin et al., 2004; Gervais et al., 2007; Martin et al., 2007; Cohen et al., 2015; Frederick et al., 2016; Fourcaud-Trocmé et al., 2019). Second, across human and nonhuman animal models, neocortical beta oscillations are strongly linked to reward processing, which is critical for learning through reinforcement (Cohen et al., 2007; Cohen et al., 2011; Kawasaki and Yamaguchi, 2013; HajiHosseini and Holroyd, 2015; HajiHosseini et al., 2020; Voloh et al., 2020; Chernyshev et al., 2023; Iturra-Mena et al., 2023; Hoy et al., 2024; Koloski et al., 2024a,b, 2025; Xiao et al., 2024). Frontal cortical beta oscillations have been observed before arrival at the goal (Xiao et al., 2024) or after the outcome at the goal has been revealed (Cohen et al., 2007; HajiHosseini and Holroyd, 2015; HajiHosseini et al., 2020; Voloh et al., 2020; Iturra-Mena et al., 2023; Xiao et al., 2024; Koloski et al., 2025), which supports the hypothesis that beta oscillations are network states supporting reward outcome processing to guide reinforcement learning (Marco-Pallarés et al., 2015). Third, we recently found learning-related changes in the power of prefrontal cortical beta oscillations at goal locations in spatial navigation tasks (Wang et al., 2026).

The dynamics of beta during initial task acquisition remain poorly understood. Furthermore, it is unknown how neocortical beta dynamics during initial task acquisition relate to hippocampal markers of learning. We therefore examined hippocampal–prefrontal cortical beta during initial task acquisition and its relationship with changes in hippocampal SWRs. To connect learning-related dynamics with known reward processing correlates of beta, we then determined how hippocampal–neocortical beta relates to task-specific variables such as reward outcome and goal choice.

## Materials and Methods

### Animals

Long–Evans rats (Charles River Laboratories, 12 males, 7–12 months) were used for the study. All procedures were approved by the university’s Institutional Animal Care and Use Committee, according to the guidelines of the Association for Assessment and Accreditation of Laboratory Animal Care.

### Pretraining

To accustom animals to exploring and drinking liquid reward (70% evaporated milk, Carnation, with 5% sucrose), we first conducted open field training with droplets scattered in the field to encourage exploration. This was done for 3 d, with each session lasting 10–20 min. Animals were then food-restricted to maintain 85–90% of their baseline weight and trained to run on an elevated linear track for reward for three 10 min sessions per day over 3 d.

### Maze apparatus

All tracks and mazes were constructed from acrylic (black DP-9, 8 cm wide, 4 cm tall walls, 76 cm above floor level). Reward delivery in all tasks was automated. Infrared sensors were located at the ends of the maze arms to detect the rat’s arrival and to trigger the reward. On rewarded trials, reward delivery was triggered at the time of goal entry. Reward was delivered by a syringe pump (100 μl at 20 ml/min, NE-500, New Era Pump Systems). The length of each arm of the Y maze was 84 cm (center to goal).

### Neural implants

Animals were chronically implanted with neural recording devices (Voigts et al., 2020) with up to 32 adjustable tetrodes. Up to 16 tetrodes targeted the CA1 region of the left hippocampus (AP −3.8, ML −2.75 relative to bregma), and up to 16 tetrodes targeted both hemispheres of the prefrontal cortex (PFC) (AP +3.5, ML ±1.5 relative to bregma and −2.5 to −3.5 DV), which corresponds to the prelimbic region (Paxinos and Watson, 2007). The PFC bundle was angled at 15° toward the midline to avoid the superior sagittal sinus. A ground screw was placed in the skull above the right cerebellum. Tetrodes were gradually adjusted into the target regions over 2–3 weeks after surgery, based on local field potential (LFP) markers and estimated depth of the tetrodes. Once the tetrodes were within the target brain regions, small adjustments of 25 μm, when necessary, were made to isolate single cells. Tetrode locations were verified at the end of the experiment. To mark the tip of the tetrode, electrolytic lesions were created for each tetrode (100 μA, 2 s). Nissl staining was used to verify the location of the lesion was within the intended area.

One male animal was implanted with electrodes (100 μm, formvar-coated stainless steel) targeting the left OB and PFC. Two electrodes (separated by 1.5 mm) were implanted in the OB (+8.5 anterior to bregma, 1.5 mm lateral to midline and 4 mm from dorsal surface) and two (separated by 1 mm) in PFC (+3.5 anterior to bregma, 1.5 lateral and −2.5 to −3.5 deep).

### Retraining

Five days prior to the main experiment, animals were food-restricted to 85–90% of their baseline weight and retrained on the linear track for 2 d.

### Maze tasks

We trained two groups of animals on Y-shaped mazes. One group (*n* = 5) was trained to perform a continuous alternation task, while the other group (*n* = 6) was trained to perform a binary multiarmed bandit task, which we refer to as the probability task. Animals were trained over 5 consecutive days, with four maze sessions (10 min each) per day. Maze sessions were separated by 45 min rests.

For the continuous alternation task, the animal needed to visit goal locations in a specific sequence to be rewarded on every trial. There were three goal locations in each maze, which were wells at the end of the arms. The animal needed to alternate visits between the left and right goals (L and R) via the center goal (C), i.e. C, L, C, R, C, L, C, R, …, consistent with previously described spatial alternation tasks (Frank et al., 2000; Kim and Frank, 2009; Jadhav et al., 2012). For example, if the rat was at the center goal, it would be rewarded next at the left goal only if it had previously visited the right goal. Similarly, if the rat was at the center goal, it would be rewarded next at the right goal only if it had previously visited the left goal. If the rat was at the left or right goals, it would be rewarded only by going to the center goal. For the probability task, each of the three goals of the Y maze had a different likelihood of delivering reward when visited (25, 50, and 75%). Once the animal had visited a goal, it had to choose between the two other goals. Repeated visits to the same goal did not yield further opportunities to claim a reward.

### Data recording

Data were collected using the Trodes data acquisition system (SpikeGadgets). Neural data were recorded using a 128-channel digitizing headstage. The headstage was tethered via a commutator to the main data acquisition system. The commutator and tether were supported by a passive suspension system. Raw data were recorded at 30 kHz. LFP (<300 Hz) was extracted at 1,500 Hz. The recording system also controlled reward delivery with its scripting function.

### Behavioral analysis

Performance on the alternation task was calculated as the mean proportion of goal visits that followed the correct alternation pattern. For the probability task, we calculated the proportion of visits to the goal with higher reward probability on the next trial. For example, if the animal had previously visited the 25% goal, the next choice is counted as correct if the animal visited the 75% goal rather than the 50% goal.

### Spectral analysis

We computed beta power for each brain region using a continuous wavelet transform (CWT). For the LFP from each tetrode, we first applied CWT using the generalized Morse wavelet (*γ* = 4, *β* = 7.5) to extract time-resolved spectral power for 0.5–130 Hz in 1 Hz steps. This was done using the GhostiPy package (Chu and Kemere, 2021). The power in the beta band (15–30 Hz) isolated from the CWT spectrogram was then calculated. We found a consistent transient increase in beta power occurring after goal entry. Goal entry was defined as the time when the animal triggered the infrared sensor at the goal. To calculate the burst of beta power after goal entry, we identified a 500 ms window centered on the time of the peak of the beta burst for each day for each animal. We calculated the mean power in the 500 ms window, and to control for changes in baseline beta power, we subtracted the beta power of a baseline period, defined as the 500 ms before goal entry.

### SWR detection

SWRs were identified based on previous methods (github.com/Eden-Kramer-Lab/ripple_detection; Kay et al., 2016). We first filtered out artifacts due to LFP noise by excluding time intervals in which the LFP signal magnitude exceeded 10 standard deviations of values across all tetrodes. LFP from tetrodes in the dorsal CA1 region (oriens and pyramidale layers) was filtered in the ripple frequency band (150–250 Hz). The median of the smoothed envelope (Gaussian kernel *σ* = 4 ms) was calculated. We used a threshold of 2 standard deviations for detecting SWRs, where the peak value of the ripple-frequency-filtered signal needed to exceed 2 standard deviations to be classified as a ripple. In addition, a detected event needed to have a minimum duration of 15 ms and separation of 50 ms. Only SWR events occurring at speed <4 cm/s and at the goal locations were included. The SWR size was calculated as the mean standard deviation above the mean ripple-frequency-band-filtered signal for the duration of the SWR. The SWR rate was calculated for the first 10 s after goal entry. Goal visits shorter than this duration were excluded from the calculation. The 10 s interval is based on the behavior of the animal at the goal, where it typically remains immobile to consume the reward.

### Experiment design and statistical analyses

We used linear mixed-effects models (LMMs) to account for variance explained by fixed as well as random effects (from individual animals). We report the marginal *R*^2^ value, which is the proportion of variance explained by the fixed effect, and the conditional *R*^2^ value, which is the proportion of variance explained by the fixed and random effects (Nakagawa and Schielzeth, 2013). Multiple comparisons were corrected using the Benjamini–Hochberg method. We used Python packages for statistics: SciPy (scipy.org) and statsmodels (statsmodels.org) for LMMs. We used pingouin (pingouin-stats.org) for ANOVA. Plots were made using Matplotlib (matplotlib.org).

Day averages are mean values (beta power, SWR rate or size, reward probability, or goal visit probability) of all trials on each day. Session averages are mean values of all trials within each of the four sessions per day. To normalize per-trial values within each day, we converted values for each trial into a *z* score by subtracting the mean and dividing by the standard deviation of all trials of the day. The trial values could then be grouped by goal location or sessions. This within-day normalization still allows us to identify differences between goal locations or across sessions within the same day while accounting for changes between days.

## Results

### Spatial navigation tasks with distinct rules

To characterize neocortical beta dynamics during initial task acquisition, we chose spatial learning tasks because rats require minimal shaping or habituation for navigating space. This also complements our understanding of neocortical beta dynamics from operant conditioning tasks, which have been the task of choice in prior studies (Iturra-Mena et al., 2023; Koloski et al., 2024a,b, 2025). We trained two groups of rats over 5 d, with four 10 min sessions per day separated by 45 min rests, on either a Y maze with a deterministic or a probabilistic reward rule. We recorded local field potentials simultaneously in dorsal CA1 of the hippocampus and prelimbic region (referred to as PFC). By comparing the two groups, we aimed to dissect which aspects of the dynamics could be attributed to general learning-related changes versus rule-specific differences.

Given that prior studies have shown strong correlates of frontal cortical beta with reward likelihood and outcome (Cohen et al., 2007; HajiHosseini and Holroyd, 2015; HajiHosseini et al., 2020; Voloh et al., 2020; Iturra-Mena et al., 2023; Xiao et al., 2024; Koloski et al., 2025), we designed our tasks to vary along these dimensions. The deterministic and probabilistic reward rules were chosen to dissociate the animal's certainty about the optimal goal visit pattern from the certainty of reward delivery for each goal visit. One group (*n* = 5) was trained on a continuous alternation task in which the animals learned to visit the three goals of the Y maze in an alternation sequence (C to L to C to R and repeat…; Fig. 1*A*, left). In this deterministic reward task, each visit was rewarded with certainty if the animal learned the sequence of goal visits; thus, the maximum reward rate was 100% of all goal visits. The second group (*n* = 6) was trained on a binary multiarmed bandit task, where each of the three goals of the Y maze had a different probability of delivering reward (25, 50, and 75%; Fig. 1*A*, right). In this task, the animals had to learn to visit, back and forth, the two goals with the highest probabilities to maximize reward (50% to 75% to 50% and repeat…). However, even if the animal followed this pattern, the outcome of each goal visit remained uncertain, and the maximum reward rate for the probability task was 62.5% of all goal visits.

**Figure 1.**
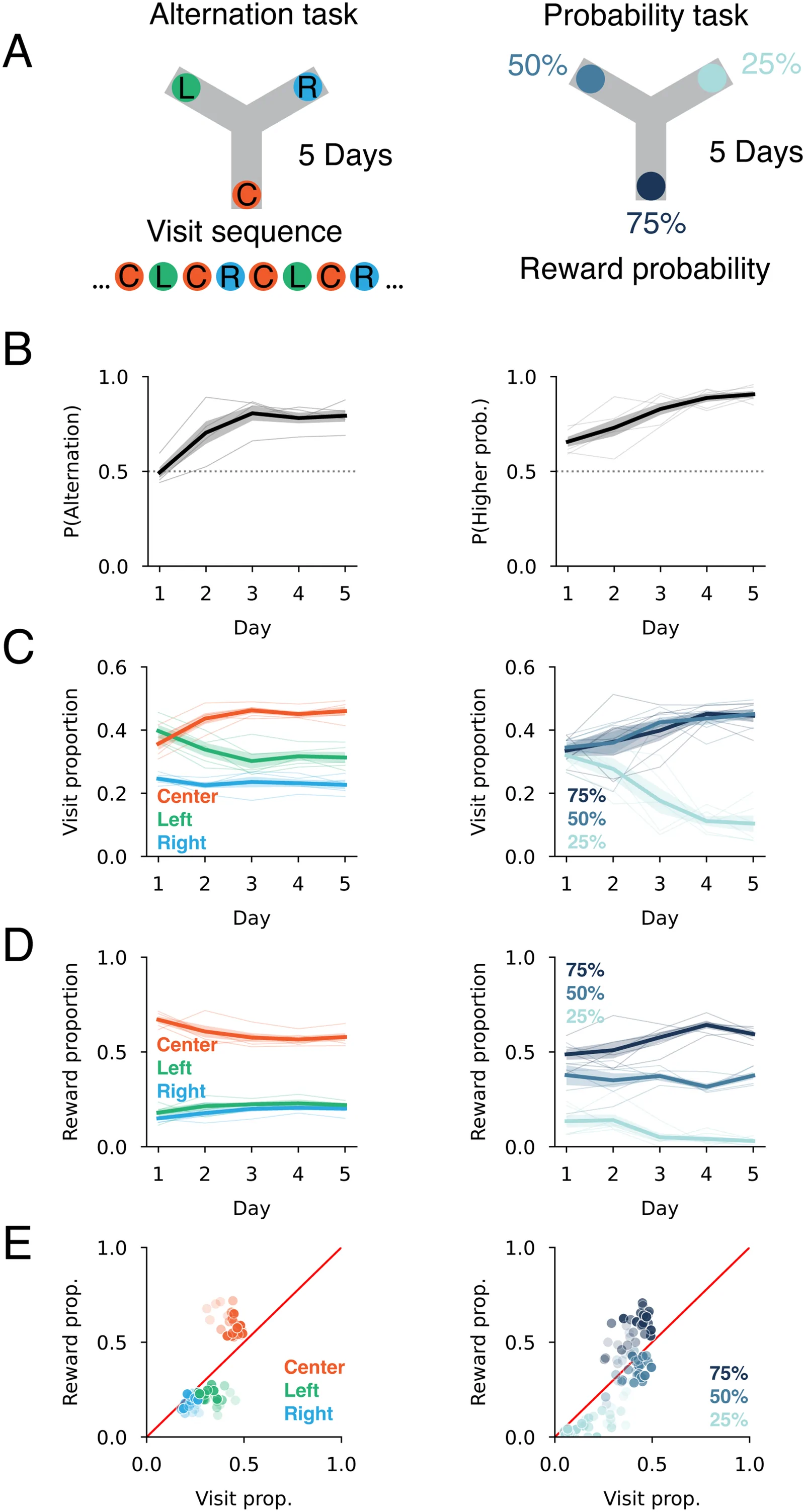
Two spatial navigation tasks with distinct rules. ***A***, Y maze continuous alternation task (left): the animal needs to learn to alternate visiting two of the three goal locations via the center goal location. Y maze probability task (right): each of the three maze arms has 25, 50, or 75% reward probability. After visiting the goal location at one arm, the animal needs to visit one of the two other arms. Each animal was trained for four 10 min sessions per day over 5 d. ***B***, Proportion of trials matching the expected goal visit pattern for alternation (left) and probability (right) rules over the 5 d of training. For the probability task, the optimal choice is to always choose the goal with the higher probability. ***C***, Proportion of visits to each goal for the alternation (left) and probability (right) tasks for each day. ***D***, Proportion of rewards received at each goal location for the alternation (left) and probability (right) tasks for each day. ***E***, Scatter of reward proportion (***D***) versus visit proportion (***C***). Colors indicate goal location and shading intensity indicates day.

Over the 5 d of training, animals in both groups learned their respective tasks. Across days of training, animals showed an increase in the proportion of expected visit patterns for each rule (Fig. 1*B*). As expected, the two tasks had distinct reward yields. In the alternation task, 77 ± 3.5% of trials were rewarded in the last days of training, whereas in the probability task, 56 ± 1.8% of trials were rewarded (Wilcoxon rank-sum test *p* = 0.0062). Animals in both groups showed choices that maximized reward rate over days of training. In the alternation task, the animals visited the center arm of the Y maze at approximately twice the rate of the side arms (Fig. 1*C*, left). For the probability task, the animals learned over days to visit the two goals with the highest reward probabilities (50 and 75%) while avoiding the goal with the lowest reward probability (25%; Fig. 1*C*, right). In each task, the proportion of reward received at each goal differed, remaining stable across days for the alternation task, while converging toward higher proportions for the two high-likelihood goals in the probability task (Fig. 1*D*). Over the days of training, the goal choices in both tasks became more closely matched to the proportion of reward received at each goal (Fig. 1*E*). The animals became more certain about their choice of goals to visit in both tasks over time, despite inherent differences in certainty about the reward outcome of each goal visit between the tasks.

### Goal-entry beta power increases with training in PFC but not CA1

We next examined beta oscillations when the animal reached the goal, given the importance of goal outcome processing in learning and the prevalence of both beta oscillations and SWRs during this period. We characterized beta dynamics in PFC, since less is known about goal-related beta dynamics in this region of the rodent frontal cortex despite its importance in reward learning across task domains (Dalton et al., 2016; Maharjan et al., 2018; Verharen et al., 2020). Prior work on reward-related frontal cortical beta oscillations has primarily investigated the orbitofrontal cortex (OFC; Koloski et al., 2024a,b, 2025) and anterior cingulate cortex (ACC; Iturra-Mena et al., 2023).

We previously found that beta oscillations occurred as animals visited goal locations in spatial navigation tasks (Wang et al., 2026). Consistent with our previous findings, we saw increases in beta frequency power in PFC and CA1 LFP relative to goal entry. The increase in beta power is not explained by gross movement of the animal since the animal remains immobile after goal entry (Fig. 2*A*, speed profile). However, we noticed a strong experience-related pattern for the PFC burst that occurred immediately after goal entry. On the first day of training, there was low PFC power in the beta frequency range within the first second after goal entry (Fig. 2*A*). However, on Day 5 of training, we saw a clear burst of beta (Fig. 2*B*). We quantified the increase in beta power after goal entry relative to a 500 ms window before goal entry. This normalization accounts for changes in the baseline beta power on each tetrode across sessions or days. The goal-entry PFC beta power increased over days (Fig. 2*C*; Extended Data Fig. 2-1*A*) of training and with improved performance (Fig. 2*D*), for both the alternation and probability tasks. CA1 beta power did not show a significant change across days (Fig. 2*E*; Extended Data Fig. 2-1*B*) and was negatively correlated with performance for the alternation task but not the probability task (Fig. 2*F*).

**Figure 2.**
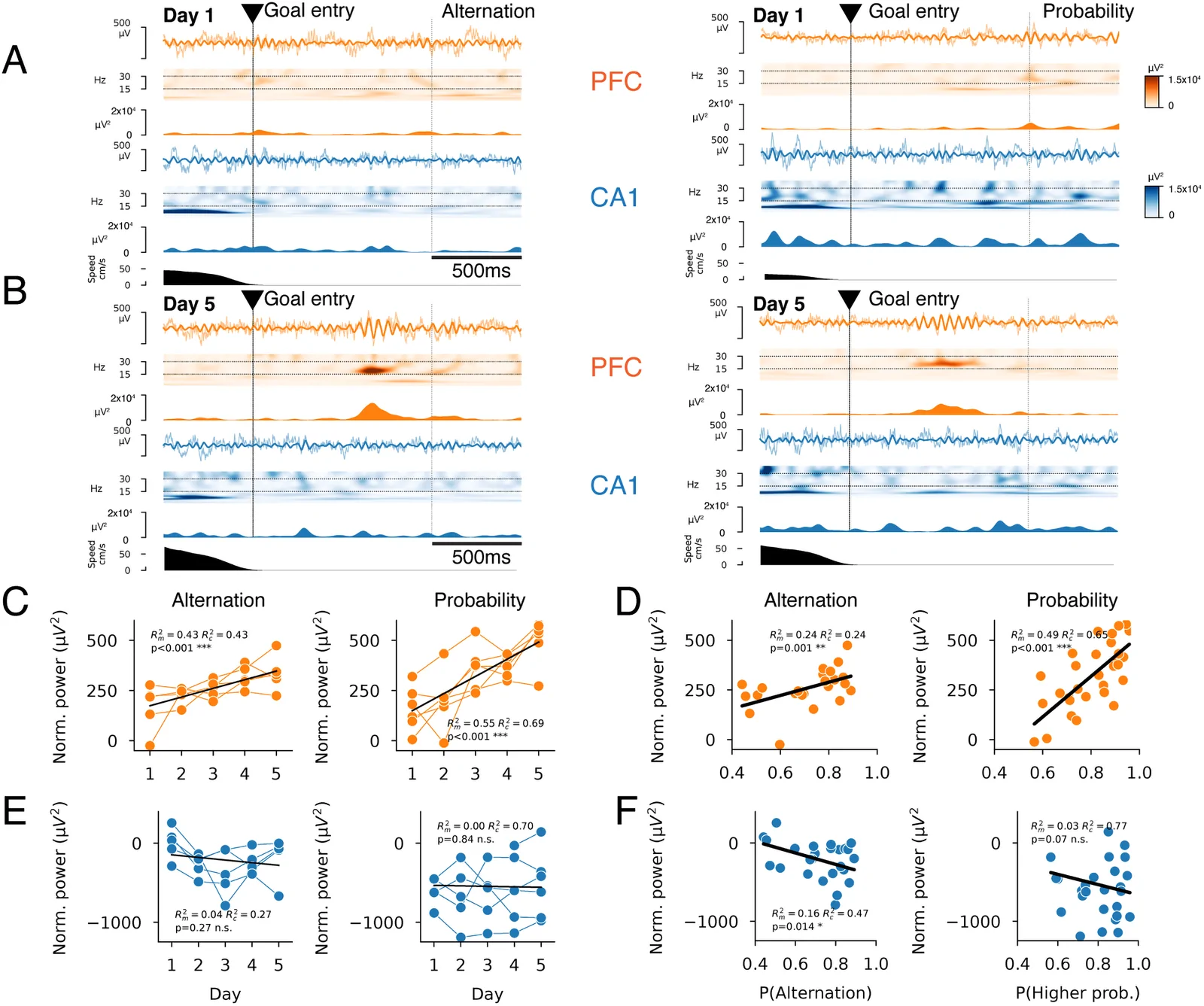
PFC but not CA1 goal-entry beta power increases over days of training. ***A***, Example goal-entry-aligned LFP traces for PFC (orange) and CA1 (blue) for Day 1 for the alternation task (left) and probability task (right). For each brain region, the raw and beta-frequency-filtered LFP is shown above the power spectrum from a CWT. The speed of the animal is shown in the bottom row. The solid vertical line indicates the time of goal entry. The dotted vertical line marks 1 s after goal entry. Time scale bar indicates 500 ms. ***B***, Example goal-entry-aligned LFP traces for PFC (orange) and CA1 (blue) for Day 5 for the alternation task (left) and probability task (right). A burst of beta occurs after goal location entry on Day 5. ***C***, PFC goal-entry beta power across 5 d of training. PFC power is the mean beta frequency power within a 500 ms window spanning the peak (see Materials and Methods) after goal entry. Data points for each animal are joined by lines. An LMM with fixed effect (days) and random effect (animal) was used to fit the data. The line of best fit is derived from the fixed effect component of the model. Alternation: *β* = 43.24, SE = 10.20, *z* = 4.24, *p* < 0.001. Probability: *β* = 85.51, SE = 11.92, *z* = 7.17, *p* < 0.001. Mean goal-entry-aligned power shown in Extended Data Figure 2-1. Control for volume conduction between OB and PFC in Extended Data Figure 2-2. PFC and CA1 power spectral density for maze and goal periods shown in Extended Data Figure 2-3. Goal-entry speed profile shown in Extended Data Figure 2-4. ***D***, PFC beta power versus performance in each task for each day. Performance is the proportion of trials matching the rule of the task (Fig. 1*B*). Alternation: *β* = 332.39, SE = 104.01, *z* = 3.20, *p* = 0.001. Probability: *β* = 1020.09, SE = 167.51, *z* = 6.09, *p* < 0.001. ***E***, CA1 goal-entry power in the beta frequency across 5 d of training. An LMM with fixed effect (days) and random effect (animal) was used to fit the data. Alternation: *β* = −33.19, SE = 30.16, *z* = −1.10, *p* = 0.27. Probability: *β* = −6.15, SE = 31.08, *z* = −0.20, *p* = 0.84. ***F***, CA1 beta power versus performance in each task for each day. Alternation: *β* = −732.12, SE = 299.39, *z* = −2.45, *p* = 0.014. Probability: *β* = −652.76, SE = 357.45, *z* = −1.83, *p* = 0.068.

10.1523/ENEURO.0048-26.2026.f2-1 Figure 2-1 **Goal entry PFC beta power increases across days of training.**
PFC beta power aligned to goal entry. Colored lines show the median for each day for the alternation (left) or probability (right) tasks. The power was normalized by subtracting the baseline value from the 500 ms window before goal entry. We calculated the median to show that the trend was not driven by large values.CA1 beta power aligned to goal entry. Colored lines show the median for each day for the alternation (left) or probability (right) tasks. We note that prior to goal entry, there is power from theta oscillations (∼7-8 Hz). The first harmonic of theta introduces power in the ∼14-16 Hz range, which overlaps with the beta filtering range. Thus, the baseline period has higher power than the post-goal-entry period, leading to negative normalized CA1 beta power.Mean peak time of PFC beta bursts for each day for the alternation (left) or probability (right) tasks. Alternation: β = −0.021, SE = 0.032, z = −0.65, p = 0.52. Probability: β = −0.016, SE = 0.013, z = −1.23, p = 0.22. Download Figure 2-1, TIF file.

10.1523/ENEURO.0048-26.2026.f2-2 Figure 2-2 **PFC and OB beta oscillations are independent.**
Example LFP and beta frequency filtered traces for two OB (green) and PFC (orange) electrodes.Beta power correlation (Pearson’s) between pairs of OB and PFC electrodes.Arrangement of PFC tetrodes along the anterior-posterior axis. Each tetrode is housed in a 211μm tubing. The entire tubing bundle spans approximately 2.3 mm along the anterior-posterior axis.Normalized goal-entry beta power for the alternation task group for tetrodes along the anterior (0) – posterior (1) axis. Each tetrode is assigned a relative location along the anterior-posterior axis denoted by 0 (anterior) and 1 (posterior). LMM with fixed effect (A-P location) and random effect (animal): β = −8.39, SE = 29.64, z = −0.28, p = 0.78.Normalized goal-entry beta power for the probability task group. LMM with fixed effect (A-P location) and random effect (animal): β = 54.86, SE = 38.51, z = 1.43, p = 0.15. Download Figure 2-2, TIF file.

10.1523/ENEURO.0048-26.2026.f2-3 Figure 2-3 **PFC beta power cannot be explained by the first harmonic of theta oscillations.**
Alternation task power spectral density for PFC (orange) and CA1 (blue) for maze (light orange) and goal (dark orange) periods. The aperiodic component was subtracted from the raw PSD to show the periodic component. The PSD is separated to show the <15 Hz or the 10-30 Hz ranges.Difference between goal and maze period PSDs (colored line) for the alternation task. The gray shading represents the 95% confidence interval for a permutation test.Probability task power spectral density for PFC (orange) and CA1 (blue) for maze (light orange) and goal (dark orange) periods.Difference between goal and maze period PSDs (colored line) for the probability task. The gray shading represents the 95% confidence interval for a permutation test. Download Figure 2-3, TIF file.

10.1523/ENEURO.0048-26.2026.f2-4 Figure 2-4 Speed relative to goal entry. Mean (±SEM) speed in a 20 second window after goal entry.Mean (±SEM) speed across days in a 5 second window after goal entry. Download Figure 2-4, TIF file.

We performed control analyses to verify PFC beta could not be explained by volume conduction from the OB or harmonics from lower frequencies. Strong beta oscillations occur in the OB (Kay et al., 1996; Kay and Freeman, 1998), raising the possibility that electrodes in PFC, which are ∼4–5 mm posterior to the OB, could detect volume conducted beta from the OB. In simultaneous recordings from OB and PFC, we found beta bursts can occur independently in each region (Extended Data Fig. 2-2*A*), and beta power was correlated within each region but not between regions (Extended Data Fig. 2-2*B*). Furthermore, we quantified the power on PFC tetrodes based on their location along the anterior–posterior axis, with the anterior-most tetrodes being closest to the OB. If PFC beta is volume conducted from OB, we would find a consistent decrease in power toward the posterior PFC electrodes. We found beta power was not correlated with distance along the anterior–posterior axis (Extended Data Fig. 2-2*C–E*). We next excluded the possibility of theta harmonics contributing to PFC and CA1 goal-entry beta power. Beta oscillations span the 15–30 Hz range, and bandpass filtering the LFP signal could include the first harmonic of theta oscillations (∼7–8 Hz). To ensure our beta quantification was not detecting the theta harmonic, we computed the power spectral density for periods when the animal was moving on the maze or immobile at the goal. As expected, we found movement periods showed higher power in the theta frequency range whereas the immobile periods had lower power (Extended Data Fig. 2-3*A*,*C*, on maze or at goal). Importantly, LFP from goal periods in PFC showed a prominent peak at ∼20 Hz without a peak at the frequency for the harmonic of theta (∼14–16 Hz; Extended Data Fig. 2-3*B*,*D*, 10–30 Hz panels).

### Complementary training-dependent changes in PFC and CA1

Hippocampal SWRs occur when animals visit goal locations and are more frequent during early stages of learning or when exposed to a new environment or task (Foster and Wilson, 2006; Cheng and Frank, 2008; Singer and Frank, 2009; Singer et al., 2013; Ambrose et al., 2016; Tang et al., 2017; Carey et al., 2019; Huelin Gorriz et al., 2023). Consistent with previous findings, we found that CA1 SWR rate (Fig. 3*A*) and size (Fig. 3*B*) decreased over days of training on both tasks, and CA1 SWR rate and size were comparable between the tasks (one-way ANOVA: SWR rate *F*_(1,50)_ = 0.398, *p* = 0.531; SWR size *F*_(1,50)_ = 0.400, *p* = 0.528). This decrease in CA1 SWR rate and size stands in contrast to the increase in PFC goal-entry beta power (Fig. 2*C*). We confirmed this inverse relationship by correlating CA1 SWR rate (Fig. 4*A*) and size (Fig. 4*C*) with goal-entry PFC beta power for each day, which showed negative correlations for both the alternation and probability tasks. This relationship was unique to PFC, as we did not find a negative correlation between CA1 goal-entry beta power and SWR properties (Fig. 4*B*,*D*). Despite differences in the reward scheme between the two tasks, the inverse correlation between PFC beta power and CA1 SWR properties was found in both tasks, which suggests these markers correspond to general, but complementary, experience-dependent changes in hippocampal–neocortical networks across learning.

**Figure 3.**
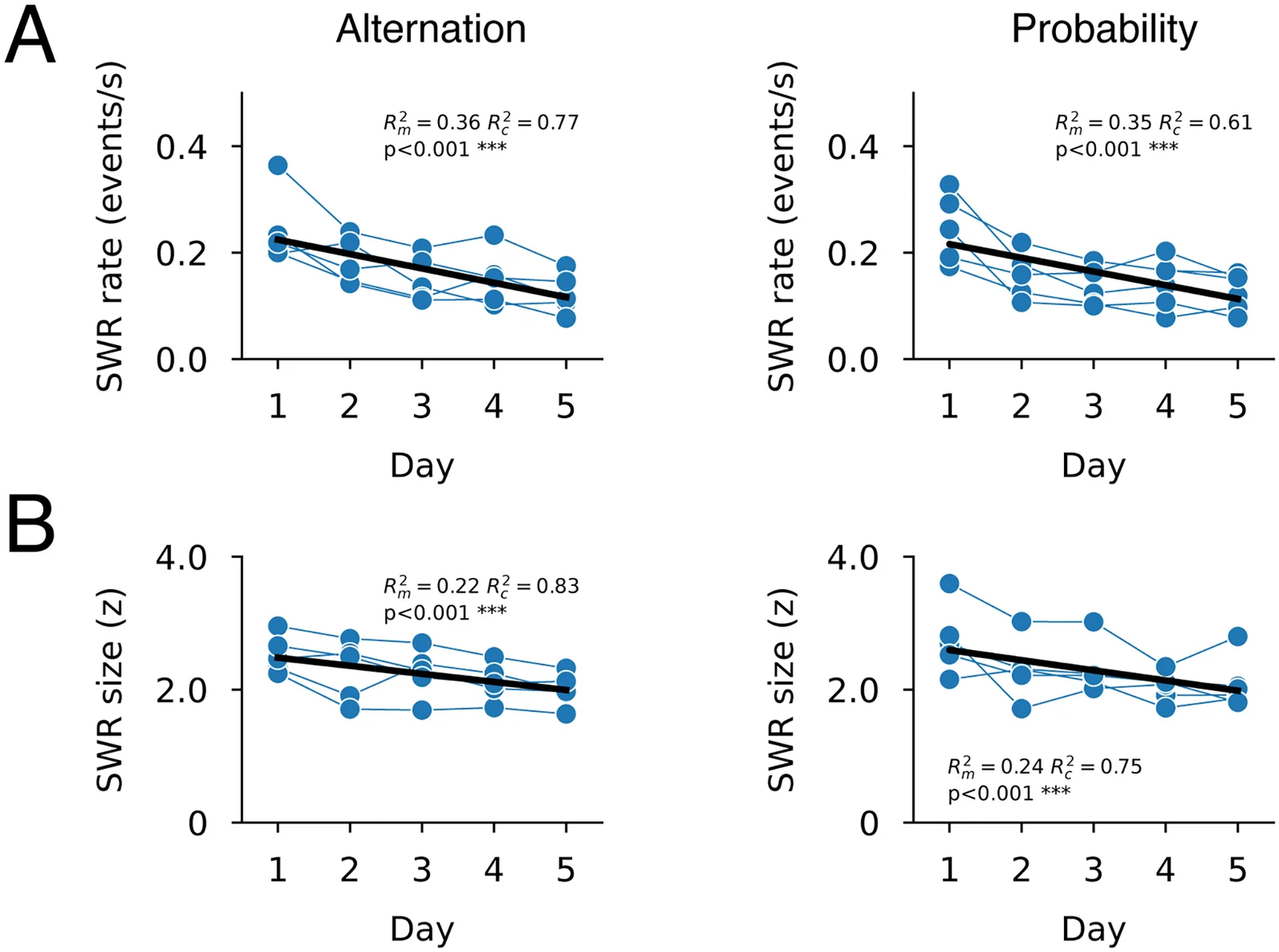
Hippocampal SWR rate and size decrease over days of training. ***A***, CA1 SWR rate across the 5 d of training. Data points for each animal are connected by lines. An LMM with a fixed effect (days) and a random effect (animal) was used to fit the data. The line of best fit is derived from the fixed effect component of the model. Alternation: *β* = −0.027, SE = 0.004, *z* = −6.18, *p* < 0.001. Probability: *β* = −0.026, SE = 0.005, *z* = −4.69, *p* < 0.001. ***B***, CA1 SWR size for the 5 d of training. Alternation: *β* = −0.122, SE = 0.022, *z* = −5.55, *p* < 0.001. Probability: *β* = −0.153, SE = 0.032, *z* = −4.78, *p* < 0.001.

**Figure 4.**
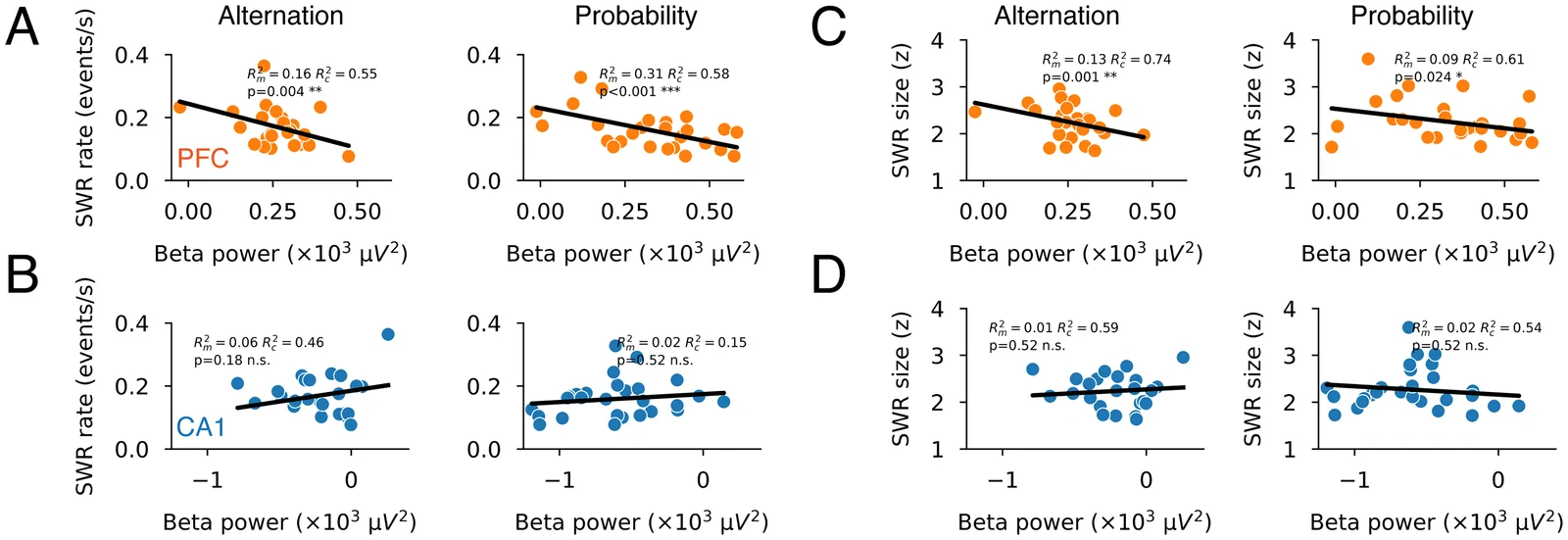
Opposing changes in PFC goal-entry beta and hippocampal SWR over days of training. ***A***, CA1 SWR rate versus PFC goal-entry beta power for 5 d of training. Each point represents the daily average for each animal. An LMM with a fixed effect (beta power) and a random effect (animal) was used to fit the data. The line of best fit is derived from the fixed effect component of the model. The marginal *R*^2^ values are shown. Alternation: *β* = −0.281, SE = 0.097, *z* = −2.88, *p* = 0.004. Probability: *β* = −0.214, SE = 0.052, *z* = −4.11, *p* < 0.001. ***B***, CA1 SWR rate versus CA1 goal-entry beta power for 5 d of training. Alternation: *β* = 0.069, SE = 0.051, *z* = 1.34, *p* = 0.18. Probability: *β* = 0.025, SE = 0.039, *z* = 0.65, *p* = 0.52. ***C***, CA1 SWR size versus PFC goal-entry beta power for 5 d of training. Alternation: *β* = −1.472, SE = 0.432, *z* = −3.41, *p* = 0.001. Probability: *β* = −0.833, SE = 0.368, *z* = −2.26, *p* = 0.024. ***D***, CA1 SWR size versus CA1 goal-entry beta power for 5 d of training. Alternation: *β* = 0.161, SE = 0.252, *z* = 0.64, *p* = 0.52. Probability: *β* = −0.178, SE = 0.276, *z* = −0.65, *p* = 0.52.

The across-day changes in PFC beta power and CA1 SWR properties could reflect familiarity or experience-dependent adaptations on the timescale of days. Alternatively, changes in beta and SWR properties could be related to performance improvements observable within each day and across days. To dissociate these possibilities, we examined the relationship between PFC beta power or CA1 SWR properties and performance across the sessions on each day. Task performance and PFC beta power increased across sessions on some days but not others (Fig. 5*A,B*,*H*,*I*). When analyzed within each day, beta power was significantly correlated with performance for both tasks (Fig. 5*C*,*J*). However, within-day SWR rate and size were not correlated with performance (Fig. 5*D*–*G*,*K*–*N*). These results suggest beta power reflects learning-related changes over sessions and days, whereas SWR properties reflect experience-dependent changes across days.

**Figure 5.**
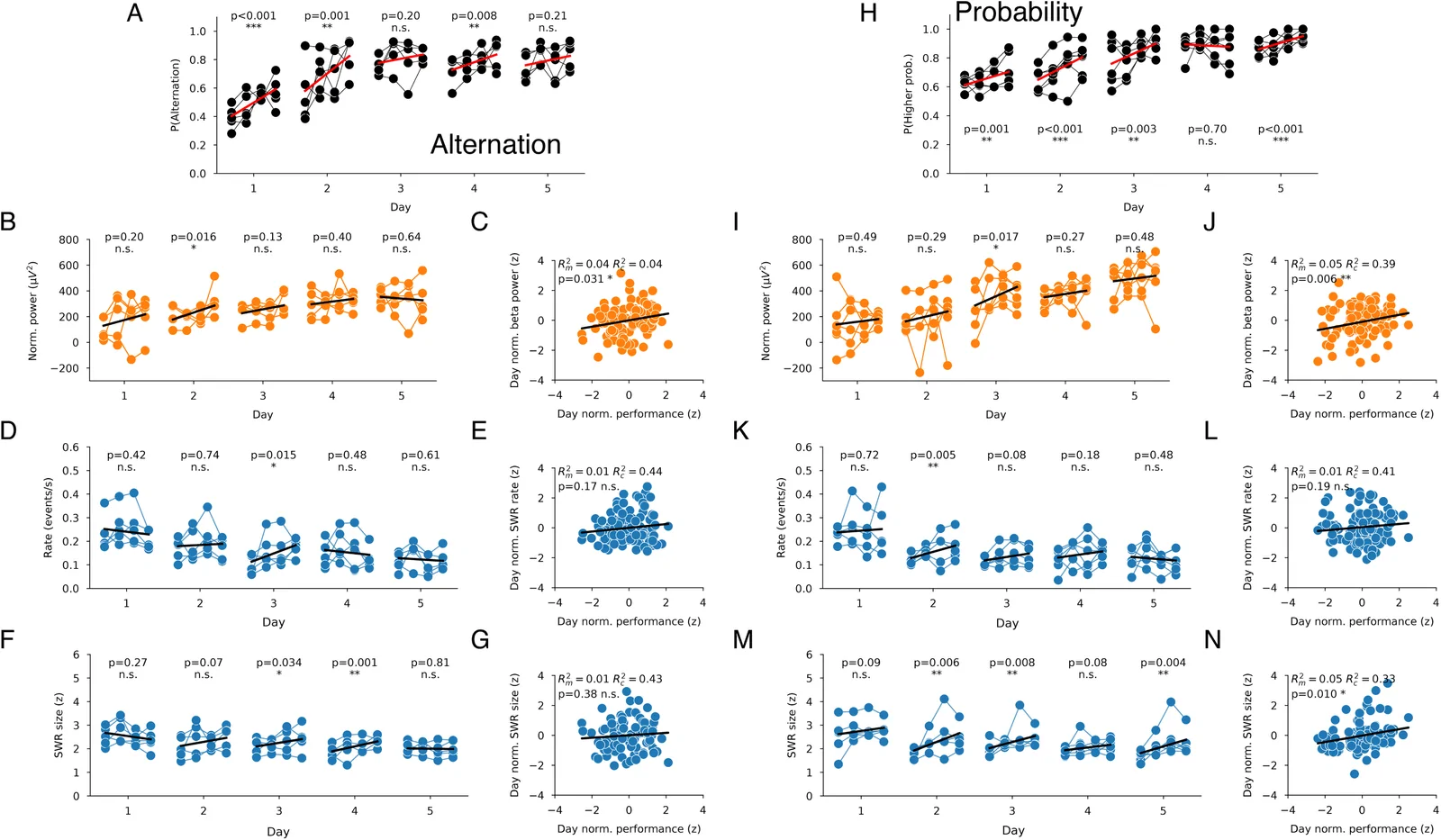
Within-day changes in behavior, PFC beta dynamics and CA1 SWR properties. ***A***, Alternation task performance across sessions. An LMM was used to determine the correlation between performance and sessions within each day. ***B***, Alternation task PFC beta power across sessions. ***C***, Correlation for within-day normalized PFC beta power and task performance. An LMM was used to determine the correlation between beta power and performance across sessions on each day, with random effects from each animal: *β* = 0.213, SE = 0.099, *z* = 2.15, *p* = 0.031. ***D***, Alternation task SWR rate across sessions. ***E***, Correlation for within-day normalized SWR rate and task performance. *β* = 0.125, SE = 0.092, *z* = 1.36, *p* = 0.17. Session-wise correlation between SWR rate and PFC beta power is shown in Extended Data Figure 5-1. ***F***, Alternation task SWR size across sessions. ***G***, Correlation for within-day normalized SWR size and task performance. *β* = 0.082, SE = 0.092, *z* = 0.88, *p* = 0.38. Session-wise correlation between SWR size and PFC beta power is shown in Extended Data Figure 5-1. ***H***, Probability task performance across sessions. ***I***, Probability task PFC beta power across sessions. ***J***, Correlation for within-day normalized PFC beta power and task performance. *β* = 0.234, SE = 0.085, *z* = 2.74, *p* = 0.006. ***K***, Probability task SWR rate across sessions. ***L***, Correlation for within-day normalized SWR rate and task performance. *β* = 0.107, SE = 0.081, *z* = 1.32, *p* = 0.19. Session-wise correlation between SWR rate and PFC beta power is shown in Extended Data Figure 5-1. ***M***, Probability task SWR size across sessions. ***N***, Correlation for within-day normalized SWR size and task performance. *β* = 0.215, SE = 0.084, *z* = 2.58, *p* = 0.010. Session-wise correlation between SWR size and PFC beta power is shown in Extended Data Figure 5-1.

10.1523/ENEURO.0048-26.2026.f5-1 Figure 5-1 **Opposing changes in PFC goal-entry beta and hippocampal SWR are gradual across day effects.**
CA1 SWR rate versus PFC goal-entry beta power. Each point corresponds to the average value for one session for one animal. A LMM with fixed effect (days) and random effect (animal) was used to fit the data. The line of best fit is derived from the fixed effect component of the model. Alternation: β = −0.200, SE = 0.050, z = −3.98, p < 0.001. Probability: β = −0.154, SE = 0.030, z = −5.19, p < 0.001.CA1 SWR size versus PFC goal-entry beta power. Alternation: β = −0.628, SE = 0.298, z = −2.11, p = 0.035. Probability: β = −0.235, SE = 0.236, z = −1.00, p = 0.32.CA1 SWR rate versus CA1 goal-entry beta power. Alternation: β = 0.026, SE = 0.026, z = 1.00, p = 0.32. Probability: β = −0.002, SE = 0.022, z = −0.08, p = 0.94.CA1 SWR size versus CA1 goal-entry beta power. Alternation: β = −0.047, SE = 0.146, z = −0.32, p = 0.75. Probability: β = −0.276, SE = 0.162, z = −1.70, p = 0.09.Normalized CA1 SWR rate versus normalized PFC goal-entry beta power. Each point represents the day-normalized session average for each animal. Alternation: β = −0.116, SE = 0.136, z = −0.85, p = 0.39. Probability: β = −0.030, SE = 0.096, z = −0.32, p = 0.75.Normalized CA1 SWR size versus normalized PFC goal-entry beta power. Alternation: β = 0.186, SE = 0.151, z = 1.23, p = 0.22. Probability: β = 0.352, SE = 0.124, z = 2.85, p = 0.004.Normalized CA1 SWR rate versus normalized CA1 goal-entry beta power. Alternation: β = −0.210, SE = 0.114, z = −1.84, p = 0.07. Probability: β = −0.139, SE = 0.106, z = −1.31, p = 0.19.Normalized CA1 SWR size versus normalized CA1 goal-entry beta power. Alternation: β = −0.004, SE = 0.143, z = −0.03, p = 0.98. Probability: β = −0.373, SE = 0.143, z = −2.61, p = 0.009. Download Figure 5-1, TIF file.

We next determined the relationship between PFC beta and SWR properties within each day. One hypothesis is that the inverse relationship between PFC beta and hippocampal SWR properties across days (Fig. 4) could reflect gradual changes in each network across learning. Alternatively, the power of PFC beta may actively regulate the occurrence of hippocampal SWRs or vice versa, which would lead to the inverse relationship across sessions within the same day. We can test these hypotheses by removing the across-day effects we found earlier (Fig. 4). If the inverse relationship remains after accounting for across-day changes, then PFC beta and SWRs may be regulated on a faster timescale as well as over days. We therefore performed the correlation across sessions on a given day before (Extended Data Fig. 5-1*A*–*D*) and after (Extended Data Fig. 5-1*E*–*H*) normalizing beta power and CA1 SWR property values within the day. This within-day normalization eliminated the negative correlation between PFC beta power and CA1 SWR properties (Extended Data Fig. 5-1, compare *A*, *B* and *E*, *F*), which is consistent with our within-day analysis showing SWR properties were not related to performance within each day. Overall, PFC goal-entry beta and CA1 SWRs are likely independent markers for gradual experience-dependent changes on the timescale of sessions and days.

### PFC goal-entry beta power does not signal outcome at the goal

In addition to identifying experience-dependent changes in mean beta power, we asked whether goal-entry beta in PFC and CA1 was correlated with other task variables. In the frontal cortex, reward period beta power correlates with reward outcome (Cohen et al., 2011; HajiHosseini and Holroyd, 2015; Chernyshev et al., 2023; Iturra-Mena et al., 2023; Xiao et al., 2024), which supports the hypothesis that beta frequency activity is a network signature for reward outcome processing. However, it is unknown whether rat PFC beta power reflects reward outcome as rat PFC spiking shows weaker reward outcome correlates compared with OFC (Sul et al., 2010; Hong et al., 2019). We therefore compared beta burst power between rewarded and unrewarded goal visits. In our experiment, the reward was triggered by the goal-entry event to immediately reveal the outcome for that goal visit. If PFC beta power was correlated with goal outcome, we would expect to find a difference in goal-entry PFC beta power between rewarded and unrewarded goal visits. Comparing the two tasks allows us to separate the two potentially confounded task variables: outcome and choice. In the alternation task, the outcome is deterministic: a correct choice to visit a goal guarantees a reward, so the outcome and the choice are linked and cannot be distinguished. In the probability task, outcome is stochastic: a correct choice to visit the highest-probability goal does not guarantee reward, so the outcome is dissociated from the choice. The probability task can test whether goal-entry beta power tracks outcome. In PFC and CA1, goal-entry beta power was similar irrespective of goal visit outcome. Although PFC beta on rewarded trials was significantly higher than on unrewarded trials when all trials were analyzed each day (Fig. 6*C*, right panel), controlling for goal location identity eliminated this difference (Extended Data Fig. 6-1*A*, right panels). This result is likely explained by differences between goals rather than outcome, which we examine in the next section. Thus, PFC beta after goal entry is unlikely to reflect the outcome at the goal.

**Figure 6.**
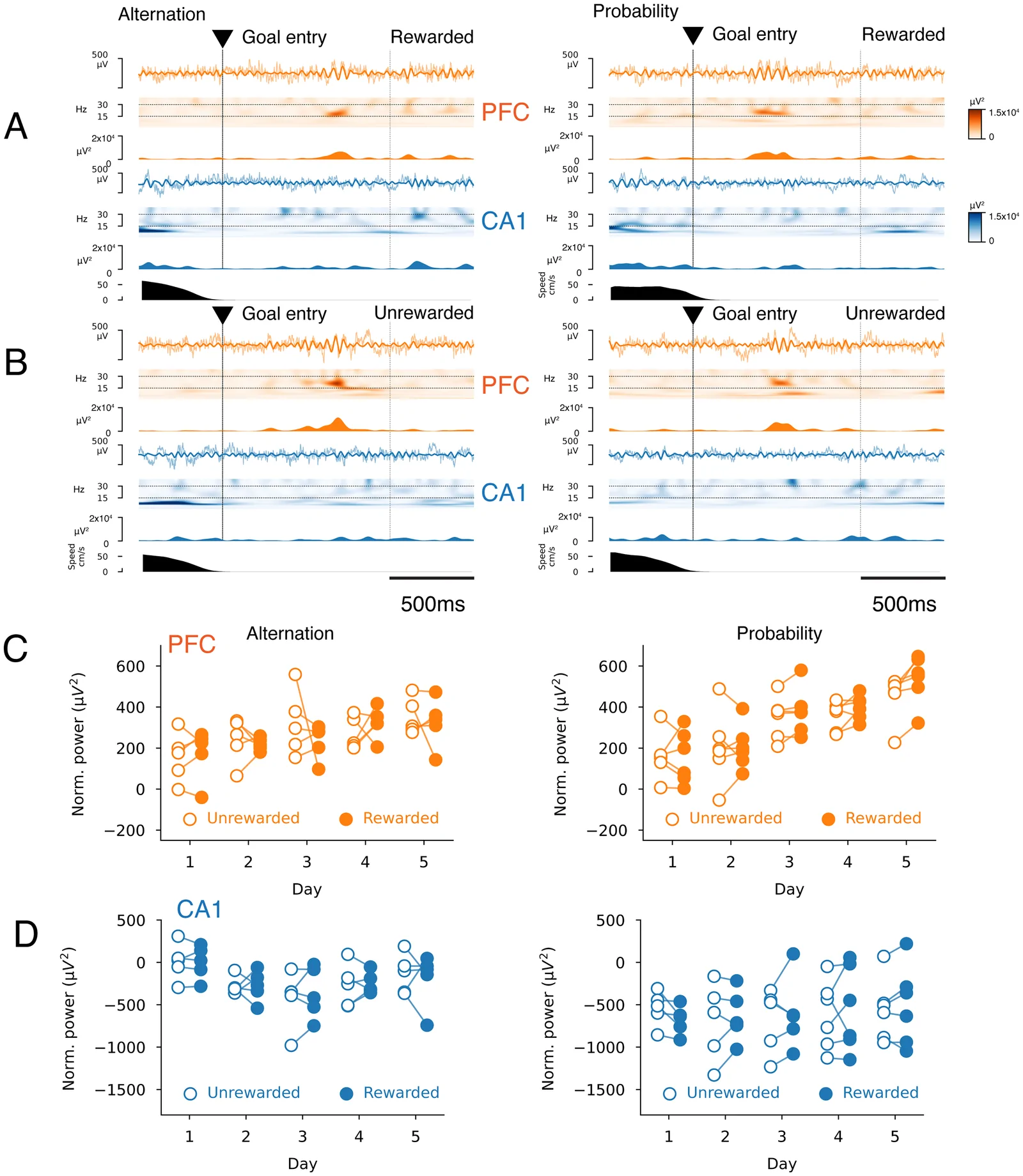
PFC and CA1 beta power is not signaling goal outcome. ***A***, Goal-entry-aligned LFP traces for PFC (orange) and CA1 (blue) for rewarded trials. Examples are from Day 5 of training. Reward was delivered at the time of goal entry. The speed of the animal is shown in the bottom row. The solid vertical line indicates the time of goal entry. The dotted vertical line marks 1 s after goal entry. Time scale bar indicates 500 ms. ***B***, Goal-entry-aligned LFP traces for PFC (orange) and CA1 (blue) for unrewarded trials. Examples are from Day 5 of training. The reward was not delivered at the time of goal entry. ***C***, Postgoal-entry PFC beta power for rewarded (filled) and unrewarded (empty) trials for each training day. Each data point represents the mean for one animal. PFC beta power did not differ between rewarded and unrewarded goal visits using a repeated-measures ANOVA for the alternation task (left: *F*_(1,4)_ = 0.06, *p* = 0.82 n.s.) but did for the probability task (right: *F*_(1,5)_ = 16.2, *p* = 0.01*). The per goal comparison is in Extended Data Figure 6-1. Goal-entry power by reward history is in Extended Data Figure 6-2. ***D***, Postgoal-entry CA1 beta power for rewarded (filled) and unrewarded (empty) trials for each training day. Each data point represents the mean for one animal. CA1 beta power did not differ between rewarded and unrewarded goal visits using a repeated-measures ANOVA for the alternation task (left: *F*_(1,4)_ = 0.02, *p* = 0.91 n.s.) and the probability task (right: *F*_(1,5)_ = 0.07, *p* = 0.83 n.s.).

10.1523/ENEURO.0048-26.2026.f6-1 Figure 6-1 **PFC and CA1 beta power for each goal location.**
Post-goal-entry PFC beta power for rewarded and unrewarded trials for each goal for each training day. Each data point represents the mean for one animal. PFC beta power did not differ between rewarded and unrewarded goal visits for each goal. Pairwise Wilcoxon rank sum test with Benjamini-Hochberg false discovery rate correction between rewarded and unrewarded trials at each goal for alternation task (left: goal 1 p = 1.0; goal 2 p = 0.75; goal 3 p = 1.0) and probability task (right: goal 1 p = 0.23; goal 2 p = 0.23; goal 3 p = 0.23).Post-goal-entry CA1 beta power for rewarded and unrewarded trials for each goal for each training day. Pairwise Wilcoxon rank-sums test with Benjamini-Hochberg false discovery rate correction between rewarded and unrewarded trials at each goal for alternation task (left: goal 1 p = 1.0; goal 2 p = 1.0; goal 3 p = 1.0) and probability task (right: goal 1 p = 0.84; goal 2 p = 0.84; goal 3 p = 0.84). Download Figure 6-1, TIF file.

10.1523/ENEURO.0048-26.2026.f6-2 Figure 6-2 **Goal entry beta power and reward history.**
Alternation task normalized goal-entry PFC beta power for rewarded or unrewarded trials relative to the last unrewarded trial. Rewarded: β = −0.008, SE = 0.034, z = −0.24, p = 0.81. Unrewarded: β = 0.113, SE = 0.061, z = 1.86, p = 0.06.Alternation task normalized goal-entry CA1 beta power for rewarded or unrewarded trials relative to the last unrewarded trial. Rewarded: β = −0.007, SE = 0.034, z = −0.21, p = 0.83. Unrewarded: β = −0.092, SE = 0.066, z = −1.40, p = 0.16.Probability task normalized goal-entry PFC beta power for rewarded or unrewarded trials relative to the last unrewarded trial. Rewarded: β = 0.009, SE = 0.030, z = 0.28, p = 0.78. Unrewarded: β = 0.016, SE = 0.033, z = 0.47, p = 0.64.Probability task normalized goal-entry CA1 beta power for rewarded or unrewarded trials relative to the last unrewarded trial. Rewarded: β = −0.027, SE = 0.031, z = −0.88, p = 0.38. Unrewarded: β = 0.006, SE = 0.034, z = 0.18, p = 0.86.Alternation task normalized goal-entry PFC beta power for rewarded or unrewarded trials relative to the last rewarded trial. Rewarded: β = 0.035, SE = 0.055, z = 0.64, p = 0.52. Unrewarded: β = −0.158, SE = 0.096, z = −1.65, p = 0.10.Alternation task normalized goal-entry CA1 beta power for rewarded or unrewarded trials relative to the last rewarded trial. Rewarded: β = −0.015, SE = 0.056, z = −0.26, p = 0.79. Unrewarded: β = 0.026, SE = 0.100, z = 0.26, p = 0.80.Probability task normalized goal entry PFC beta power for rewarded or unrewarded trials relative to the last rewarded trial. Rewarded: β = −0.066, SE = 0.034, z = −1.96, p = 0.05. Unrewarded: β = −0.034, SE = 0.035, z = −0.96, p = 0.34.Probability task normalized goal-entry CA1 beta power for rewarded or unrewarded trials relative to the last rewarded trial. Although the unrewarded trial analysis shows a significant p value, the conditional and marginal R^2^ are very low. Rewarded: β = −0.017, SE = 0.034, z = −0.48, p = 0.63. Unrewarded: β = −0.087, SE = 0.035, z = −2.52, p = 0.012. Download Figure 6-2, TIF file.

We further found that goal-entry beta power did not correlate with the history of goal visit outcomes. We compared beta power on goal visits based on the history of visits, whether previous visits were rewarded or not. We ensured the values were normalized to the power distribution for each goal. We did not find strong relationships between beta power in either PFC or CA1 and subsequent visits after rewarded or unrewarded visits, including after consecutive rewarded or unrewarded visits (Extended Data Fig. 6-2). Thus, goal-entry beta power in both CA1 and PFC is unlikely to reflect the history of goal visit outcomes.

### PFC and CA1 goal-entry beta correspond to distinct task variables

Rat orbitofrontal cortical beta power is correlated with the probability of reward for a given choice (Koloski et al., 2025). We asked how PFC or CA1 goal-entry beta power relates to reward probability, which would indicate the involvement of this network state in reward processing.

To characterize the relationship between goal-entry beta power and reward probability, we correlated the mean beta power at each goal location with the empirical reward probability based on the animal's behavior, defined as the proportion of rewarded visits to the goal location. In both of our tasks, the proportion of rewarded visits at each goal differed based on the animal's experience. We found PFC beta power was correlated with reward likelihood (Fig. 7*A*), whereas CA1 beta power was not correlated (Fig. 7*B*). This pattern was consistent for both tasks, despite differences in task rules. This result explains why, in the probability task, we found rewarded trials had higher beta power than unrewarded trials when all trials were combined but not when separated by location (Fig. 6*C*, right; Extended Data Fig. 6-1*A*, right): the higher-probability goals had more rewarded trials, which increased the average power value; this is controlled for with goal-specific comparisons.

**Figure 7.**
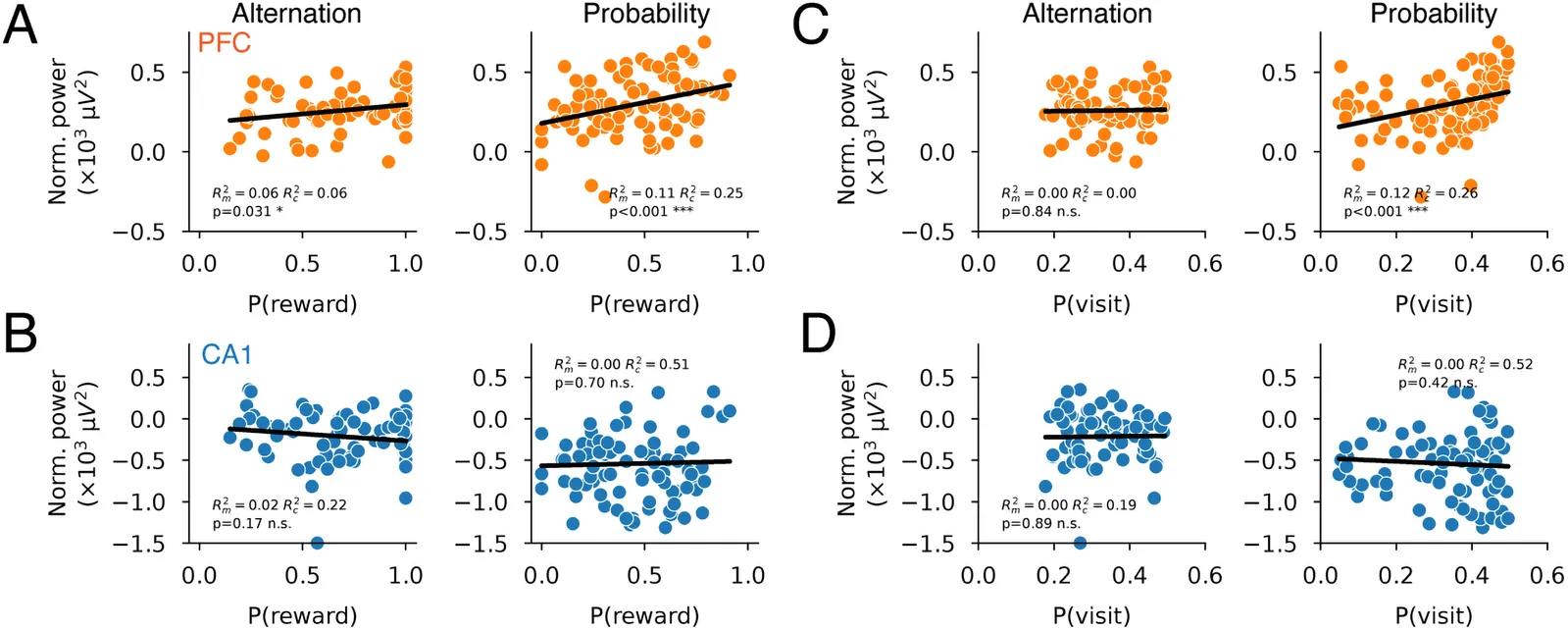
PFC beta power correlated with likelihood of reward. ***A***, Postgoal-entry PFC beta power versus reward probability at each goal for the 5 d of training. Each point represents the mean value for each goal location for each day, normalized for each day. The black line shows the line of best fit from an LMM with fixed effect (reward probability) and random effect (animal). Alternation: *β* = 0.116, SE = 0.054, *z* = 2.15, *p* = 0.031. Probability: *β* = 0.264, SE = 0.072, *z* = 3.67, *p* < 0.001. ***B***, Postgoal-entry CA1 beta power versus reward probability at each goal for the 5 d of training. Alternation: *β* = −0.171, SE = 0.126, *z* = −1.36, *p* = 0.17. Probability: *β* = 0.057, SE = 0.148, *z* = 0.39, *p* = 0.70. ***C***, Postgoal-entry PFC beta power versus proportion of visits at each goal for the 5 d of training. Alternation: *β* = 0.031, SE = 0.157, *z* = 0.20, *p* = 0.84. Probability: *β* = 0.492, SE = 0.132, *z* = 3.74, *p* < 0.001. ***D***, Postgoal-entry CA1 beta power versus proportion of visits at each goal for the 5 d of training. Alternation: *β* = 0.049, SE = 0.352, *z* = 0.14, *p* = 0.89. Probability: *β* = −0.205, SE = 0.255, *z* = −0.80, *p* = 0.42.

Interestingly, goal-entry PFC beta power was positively correlated with the proportion of visits to the goal but only for the probability task, such that more frequently visited goal locations showed higher beta power (Fig. 7*C*). In contrast, goal-entry CA1 beta power was not correlated with goal visit proportion (Fig. 7*D*). Our results suggest PFC goal-entry beta could reflect an estimate of a goal's reward likelihood (Fig. 7*A*) rather than the outcome of that visit (Fig. 6).

## Discussion

Using spatial navigation tasks with distinct reward rules, we found complementary learning-related changes in the properties of PFC beta oscillations and CA1 SWRs at goal locations. Consistent with prior findings in spatial tasks, the rate of CA1 SWRs decreased over days as the animals learned. In contrast, we found that bursts of beta in PFC occurring after the animals reached the goal showed an increase in power over days as the animals learned. We propose that the inverse relationship between these network signatures reflects gradual complementary changes in hippocampal and neocortical networks over the course of learning.

The inverse pattern sheds light on the complementary contribution of the hippocampus and neocortex to learning. Although several hypotheses have been proposed to explain the function of hippocampal SWRs, one idea is that an increase in the occurrence of SWRs reflects a response to new information that is worth capturing (van der Meer and Bendor, 2025), which is more prevalent at the start of learning when there is novelty. This increase in SWRs is thought to initiate reorganization in connected brain areas, including the neocortex, to incorporate new information into existing networks. As familiarity increases during learning, the amount of new information decreases, and we see a corresponding decrease in the rate of SWRs. In contrast to hippocampal SWRs, our results suggest beta power may signal complementary changes taking place in the neocortex during learning. During initial learning when the environment is novel, we did not observe strong goal-entry beta, but instead, beta power increased as familiarity increased. This inverse pattern mirrors what we saw with hippocampal SWRs. Low beta power may indicate an active learning state that favors the addition of new information, whereas high beta power may correspond to a postlearning state in which the availability of information has plateaued and neocortical networks have established stability.

We found that goal location beta in CA1 and PFC showed distinct correlations with task variables. In CA1, we saw beta power at goal entry was uncorrelated with reward probability for both tasks. By comparing results from a deterministic and a probabilistic reward task, we showed that PFC goal-entry beta power was correlated with reward probability and not reward outcome at each goal. This pattern shares similarity with beta dynamics in frontal cortical regions but also points to distinctions. Goal-related beta oscillations in other frontal cortical regions, such as the OFC (Koloski, Hulyalkar, et al., 2024; Koloski et al., 2025) or ACC (Iturra-Mena et al., 2023), show reward probability correlates. Notably, we found PFC beta dynamics did not correlate with the outcome at the goal. Our results provide support for regional specialization within frontal cortical regions (Shin et al., 2021; Diehl and Redish, 2023; Cunningham and Redish, 2025). Goal location spiking in PFC has stronger correlates with choice, whereas OFC spiking has stronger correlates with the reward outcome (Sul et al., 2010; Hong et al., 2019; Shin et al., 2021). Furthermore, inactivation of PFC and OFC has distinct effects on reversal learning (Dalton et al., 2016; Golebiowska and Rygula, 2017; Hervig et al., 2020). Although we have described differences in goal location beta dynamics between PFC and CA1 (Wang et al., 2026), future research is needed to determine the contribution of the goal-entry beta burst in PFC to goal-directed behaviors. Our finding that similar PFC goal-entry beta burst power occurs for rewarded and unrewarded outcomes argues for a role in expectation of the outcome rather than outcome evaluation.

Our work shows experience-dependent changes in beta dynamics in PFC; these patterns are consistent with the acquisition of task rules. These changes are complementary to SWRs in the hippocampus, pointing to coordinated changes in hippocampal–cortical networks in response to new information that warrants reorganization of existing networks (van der Meer and Bendor, 2025). The function of beta oscillations in cognition is less well understood than that of other neural oscillations (Engel and Fries, 2010; Womelsdorf et al., 2014; Spitzer and Haegens, 2017; Miles et al., 2023; Lundqvist et al., 2024). Our work raises questions about the mechanism that coordinates these complementary network changes during learning. One hypothesis is that a common novelty-driven signal may downregulate PFC beta oscillations while increasing the likelihood of hippocampal SWRs, especially during initial task acquisition. As the animal learns, the novelty signal will likely decrease, which results in stronger PFC beta oscillations and less frequent hippocampal SWRs. Alternatively, the neocortex and hippocampus may have intrinsic mechanisms for regulating learning-related changes.

Our results provide evidence that beta dynamics in hippocampal–neocortical networks at goals are distinct from known beta-modulated states in these networks. Prior work examining beta dynamics in the neocortex and hippocampus focused on cue sampling during olfactory discrimination tasks (Igarashi et al., 2014; Allen et al., 2016; Symanski et al., 2022; Jayachandran et al., 2023). During this period, there is strong coherence within the network at the beta frequency. Our results show goal-entry PFC beta is more prominent than in CA1 and has task correlates distinct from those in CA1. Thus, the goal-related beta dynamics point to a hippocampal–neocortical state that is mechanistically distinct from cue sampling. These networks are operating to subserve distinct functions for cue sampling and goal attainment.

Drawing on findings from motor control and sensory processing, our work suggests that neocortical beta may signal a stable postlearning state. In the motor cortex, beta oscillations have been associated with the state of inaction or status quo (Engel and Fries, 2010; Spitzer and Haegens, 2017). Similarly, for olfactory processing, beta power during odor sampling increases with experience (Martin et al., 2004; Gervais et al., 2007; Martin et al., 2007; Cohen et al., 2015; Fourcaud-Trocmé et al., 2019) and the transfer of learned rules (Frederick et al., 2016). Extrapolating from these concepts, we suggest that learning-dependent changes in neocortical beta oscillations signal the development of a new, postlearning status quo. Taken together, these findings provide evidence for beta oscillations being important signatures for experience-dependent changes in neocortical networks.

## Synthesis

Reviewing Editor: Adrien Peyrache, McGill University Decisions are customarily a result of the Reviewing Editor and the peer reviewers coming together and discussing their recommendations until a consensus is reached. When revisions are invited, a fact-based synthesis statement explaining their decision and outlining what is needed to prepare a revision will be listed below. The following reviewer(s) agreed to reveal their identity: NONE. Note: If this manuscript was transferred from JNeurosci and a decision was made to accept the manuscript without peer review, a synthesis may not be available.

The present study reports the observation of beta oscillations in the medial prefrontal cortex (mPFC) during reward periods. Based on electrophysiological recordings performed across different tasks, the study provides important evidence linking prefrontal beta oscillations to reward processing and learning in rodents, and how they relate to hippocampal sharp-wave ripples. The reviewers found the work interesting and potentially suitable for publication at eNeuro. However, they raised several concerns that must be addressed to warrant publication of the work.

1) While beta oscillations have been extensively studied in PFC in humans and non-human primates, most prior rodent studies have examined beta during olfactory tasks, often showing coherent beta activity between CA1 and PFC (e.g., Jayachandran et al., 2023; Allen et al., 2016; Igarashi et al., 2014; Symanski et al., 2022). In contrast, the current study reports robust PFC beta during reward delivery, which appears largely absent in CA1 (Figs. 1A-B, 5A-B) and correlates with reward-port visits and experience. This suggests that the observed PFC beta may be functionally and mechanistically distinct from previously described odor- or sniffing-related beta. A discussion explicitly addressing this distinction would clarify the novelty and broader significance of the findings.

2) Along these lines, it should be made clear whether the observed beta oscillations are locally generated (or at least are locally relevant). Specifically, given the proximity of the olfactory bulb (OB), which generates large beta field potentials (e.g. Kay et al. 2010) it is possible these reach the mPFC through volume conduction? Showing spike-field relationships, and/or a spatial profile of beta power as a function of distance to the OB could help address this important issue.

3) In Figs. 2, S1, 5, and S3, beta-band activity is quantified using raw power (µV²), whereas Figs. 6 and S4 use z-scored power normalized by daily mean and standard deviation. The rationale for switching between these approaches is not explained and should be clarified.

More importantly, comparing raw LFP power across days is problematic, as absolute power can vary due to electrode drift or impedance changes. Without within-day normalization, it is difficult to exclude the possibility that the observed increase in PFC beta reflects recording variability rather than physiological modulation.

This concern is particularly relevant for the reported enhancement in the 350-850 ms window following goal entry. Normalizing beta power within this window to a pre-goal baseline period within the same session is recommended. Reanalyzing key effects using a consistent within-day normalization procedure would substantially strengthen the conclusions.

4) In Fig. S1, beyond magnitude changes, beta enhancement appears to begin earlier across days in both tasks. Although not quantified, the time-frequency plots suggest a progressive shift in beta onset relative to goal entry. If accurate, this would be an important finding, potentially reflecting learning-related changes in anticipatory or preparatory dynamics rather than amplitude modulation alone. The authors are encouraged to quantify beta onset latency (e.g., time to significant deviation from baseline or half-max rise) across days.

5) To help clarify the above point, when was reward delivered (and consumed) relative to the time of reward entry? Is this goal-entry beta happening before, or after, the animals know whether a given trial is rewarded? This should be clarified. Relatedly, are there any changes, over days, in what the animals are doing during the post-reward window, as quantified perhaps by a measure such as average movement speed?

6) Theta oscillations (7-8 Hz) are prominent in both CA1 and PFC during spatial behavior (e.g., Siapas et al., 2005; Jayachandran et al., 2023; Igarashi et al., 2014). Activity in the lower beta range (~14-18 Hz) could reflect theta harmonics rather than a distinct beta process. Because Fig. 2A-B displays only the 15-30 Hz band, it is difficult to evaluate this possibility. Showing a broader spectrum including theta would help. Additionally, using a more conservative beta definition (e.g., {greater than or equal to}20 Hz, as in Igarashi et al., 2014) would reduce potential contamination from theta harmonics.

7) The manuscript concludes that "our work shows experience-dependent changes in beta dynamics in PFC; these patterns are consistent with the acquisition of task rules" (lines 670-671). However, because the maze was initially novel and became familiar over days, beta changes could reflect reduced novelty or general experience-dependent adaptation rather than rule learning per se. To support a rule-acquisition interpretation, beta dynamics should be shown to track behavioral performance more closely than training day. For example, does beta correlate with accuracy across sessions? Without analyses dissociating rule learning from novelty or exposure, the data demonstrate experience-dependent change but do not yet establish a specific relationship to rule acquisition.

Minor Comments

1) The manuscript reports that within-day normalization eliminates the negative correlation between PFC beta power and CA1 SWR properties, which is an interesting observation. Because animals performed four sessions per day, it would be informative to examine whether PFC beta power itself changes across the four sessions within a day over the 5-day period, in addition to the day-averaged beta in Fig. 2. Similarly, CA1 SWR properties could be analyzed at the session level across days Fig. 3. Such analyses would provide readers with a clearer sense of the timescale over which PFC beta and CA1 SWRs evolve, and would help contextualize the experience-dependent dynamics reported in the study.

2) Line 230: The phrase "Given that prior studies..." should include appropriate citations.

3) In Figs. 1A and 2A, it is unclear what the second vertical dashed line after goal entry represents. The legend should clarify whether this indicates reward delivery, the end of the 850 ms window, or another event.

4) Fig. S1B (right): The y-axis label is cut off.

5) The unit of "power" (e.g., µV²) should be indicated on the y-axis in Figs. 5 and S3.

6) "SWR size" is not defined and should be described in the Methods.

7) SWR rate was calculated over the first 10 seconds after goal entry, excluding shorter visits. The manuscript should report the average reward consumption duration and justify this threshold.

8) A fixed 500 ms window (350-850 ms after nose poke) was used to quantify beta power. A more quantitative approach would detect beta onset and offset per session, especially since beta peak timing appears to shift over days (Fig. S1).

## Author Response

We thank the reviewers for their valuable time and constructive suggestions for improving the rigor and clarity of the manuscript.

The reviewers' comments are in bold and our response are in normal font. The supplemental figures are now labelled according to the Extended Data convention for the journal.

1) While beta oscillations have been extensively studied in PFC in humans and non-human primates, most prior rodent studies have examined beta during olfactory tasks, often showing coherent beta activity between CA1 and PFC (e.g., Jayachandran et al., 2023; Allen et al., 2016; Igarashi et al., 2014; Symanski et al., 2022). In contrast, the current study reports robust PFC beta during reward delivery, which appears largely absent in CA1 (Figs. 1A-B, 5A-B) and correlates with reward-port visits and experience. This suggests that the observed PFC beta may be functionally and mechanistically distinct from previously described odor- or sniffing-related beta. A discussion explicitly addressing this distinction would clarify the novelty and broader significance of the findings.

We appreciate the recognition that the beta activity dynamics described here are distinct from those reported previously. We have clarified this point in the discussion.

2) Along these lines, it should be made clear whether the observed beta oscillations are locally generated (or at least are locally relevant). Specifically, given the proximity of the olfactory bulb (OB), which generates large beta field potentials (e.g. Kay et al. 2010) it is possible these reach the mPFC through volume conduction? Showing spike-field relationships, and/or a spatial profile of beta power as a function of distance to the OB could help address this important issue.

We agree this is an important consideration since volume conduction from the OB could potentially explain the beta oscillations in anterior frontal cortical regions. We added new analyses demonstrating the beta dynamics we found in PFC is unlikely to be explained by volume conduction.

First, we quantified beta power for each tetrode and plotted the power based on the relative position on anterior-posterior (AP) axis, as proposed by the reviewer.

Tetrodes in our recording drives are individually placed in polyimide tubes (210µm diameter) that are then bundled using a mold with a length of approximately 2.3mm (Fig. 2-2 C). We used the spatial separation between the tetrodes along the AP axis to determine whether there is a distance-dependent decrease in beta power. We assigned 0 to the anterior most tetrode and 1 to the posterior most tetrode. If there is volume conduction, we expect beta power to drop along the AP axis. We found the power remained constant. The results are reported in Fig. 2-2 D-E.

Second, we did not have high-quality single units from this dataset to perform spike-field analysis as proposed by the reviewer; instead we implanted a male rat with two electrodes in the OB and two electrodes in PFC to determine if there was volume conduction between these regions. We recorded data as the animal was freely moving in its own cage. Co-occurring beta oscillations between these regions would indicate volume conduction. However, we found independent bouts of beta in each region (Fig.

2-2A) and the beta power is most strongly correlated within each region (Fig. 2-2B).

Thus, the additional evidence indicates that PFC beta is unlikely to be explained by volume conduction alone.

3) In Figs. 2, S1, 5, and S3, beta-band activity is quantified using raw power (µV²), whereas Figs. 6 and S4 use z-scored power normalized by daily mean and standard deviation. The rationale for switching between these approaches is not explained and should be clarified.

We have changed the Fig. 7 (formerly Fig. 6) analyses to show baseline corrected power in µV² and added a better explanation for within day normalization for Fig. 6-2 (formerly Fig. S4).

More importantly, comparing raw LFP power across days is problematic, as absolute power can vary due to electrode drift or impedance changes. Without within-day normalization, it is difficult to exclude the possibility that the observed increase in PFC beta reflects recording variability rather than physiological modulation.

This is a very helpful suggestion, and we have repeated all the analyses with the baseline normalized power.

This concern is particularly relevant for the reported enhancement in the 350-850 ms window following goal entry. Normalizing beta power within this window to a pre-goal baseline period within the same session is recommended. Reanalyzing key effects using a consistent within-day normalization procedure would substantially strengthen the conclusions.

We have now used the power in the 500ms window before goal entry as the baseline as suggested. The main finding that beta power increases across days remains consistent. However, we now find the beta power is correlated with the probability of reward in both tasks but the correlation with the probability of visits is only significant for the probability task. CA1 beta power was not correlated with reward probability or visit likelihood. We have updated the text with the implications of this change.

For CA1, this baseline subtraction correction produces negative values since before goal entry, the animal is moving and there is power in the beta band due to harmonics from theta. However, our main finding remains. CA1 power during the post goal entry period does not have the same task correlation as PFC.

4) In Fig. S1, beyond magnitude changes, beta enhancement appears to begin earlier across days in both tasks. Although not quantified, the time-frequency plots suggest a progressive shift in beta onset relative to goal entry. If accurate, this would be an important finding, potentially reflecting learning-related changes in anticipatory or preparatory dynamics rather than amplitude modulation alone. The authors are encouraged to quantify beta onset latency (e.g., time to significant deviation from baseline or half-max rise) across days.

This is another potentially interesting observation. However, we did not find a statistically significant change in the peak of the burst relative to goal entry (Fig. 2-1). We quantified the half rise and the peak for each animal over days, but did not find a significant trend.

5) To help clarify the above point, when was reward delivered (and consumed) relative to the time of reward entry? The reward was delivered as soon as the animal entered the goal. The infrared beam break triggers the reward delivery immediately. In response to minor comment 5, we quantified the animal's movement at the goal. Typically, there is an immobility period of approximately 10 s before movement starts again. In the current experiments, we did not measure reward consumption but used the movement as a proxy since rats will disengage from the goal delivery device and look around after consuming the reward before leaving the goal.

Is this goal-entry beta happening before, or after, the animals know whether a given trial is rewarded? This should be clarified.

In this experiment, the beta occurred after the reward outcome was confirmed. Because beta power did not differ between rewarded or unrewarded trials (Fig. 6), it is unlikely reward outcome alone can explain the beta dynamics. Further dissecting the function of PFC beta is an active area of research in the lab.

Relatedly, are there any changes, over days, in what the animals are doing during the post-reward window, as quantified perhaps by a measure such as average movement speed? To better convey the behavior at the goal, we added the speed profile to the LFP examples (Fig. 2 and Fig.6), which show the animal is immobile in the 1.5 seconds after goal entry. To understand if there are other behavioral change after the 1s post-goal entry interval, we quantified the animal's speed over days as suggested. The animal's behavior is similar within several seconds after goal entry across days (Fig. 2-4). Typically, the animals remain at the goal for at least 10s (Fig. 2-4).

6) Theta oscillations (7-8 Hz) are prominent in both CA1 and PFC during spatial behavior (e.g., Siapas et al., 2005; Jayachandran et al., 2023; Igarashi et al., 2014). Activity in the lower beta range (~14-18 Hz) could reflect theta harmonics rather than a distinct beta process. Because Fig. 2A-B displays only the 15-30 Hz band, it is difficult to evaluate this possibility. Showing a broader spectrum including theta would help. Additionally, using a more conservative beta definition (e.g., {greater than or equal to}20 Hz, as in Igarashi et al., 2014) would reduce potential contamination from theta harmonics.

The reviewer raised another important consideration. We performed additional analyses to show the PFC beta is unlikely to be explained by theta harmonics.

First, we extended the frequency range of the spectrograms in Figs. 2 and 6 to span 0-40Hz. In these examples, PFC beta oscillations were visible from the raw LFP and did not occur together with lower frequency oscillations.

Second, we calculated the power spectral density (PSD) for periods when the animal was moving or when the animal was at the goal and not moving. We subtracted the aperiodic component to highlight the periodic components at theta, its harmonic and beta. We also calculated the difference in PSD between maze and goal periods. During movement, we can clearly see theta (~7Hz) and its harmonic (~14Hz) in CA1 (Fig. 2-3 A and C, light blue). However, those are not present at the goal when the animal was immobile (Fig. 2-3 A and C, dark blue.). For PFC, we saw higher power in the <10Hz range during movement on the maze (Fig. 2-3 A and C, light orange), but this drops during periods at the goal (Fig. 2-3 A and C, dark orange).We see a prominent increase in the ~20Hz beta (Fig. 2-3 B and D). In PFC, there is low power in the frequency range for the first theta harmonic (~14Hz). Therefore, the 15-30Hz frequency range can be used to isolate goal entry PFC beta without the concern that the first theta harmonic is contaminating the power.

These PSDs show that at goals, when the animal was immobile, we do not have strong theta or its first harmonic. These quantifications reflect the raw LFP examples (Fig. 2 and 6), which show clear beta bursts but without lower frequency oscillations in the theta range.

7) The manuscript concludes that "our work shows experience-dependent changes in beta dynamics in PFC; these patterns are consistent with the acquisition of task rules" (lines 670-671). However, because the maze was initially novel and became familiar over days, beta changes could reflect reduced novelty or general experience-dependent adaptation rather than rule learning per se. To support a rule-acquisition interpretation, beta dynamics should be shown to track behavioral performance more closely than training day.

We agree with the reviewer, this is an important distinction we need to clarify. We added additional across session analyses (Fig. 5).

For example, does beta correlate with accuracy across sessions? Without analyses dissociating rule learning from novelty or exposure, the data demonstrate experience- dependent change but do not yet establish a specific relationship to rule acquisition.

Yes, when we performed the across session analysis as suggested, we found a significant correlation between PFC beta power and performance (Fig. 5). These results indicate the beta power is related to performance on a session-by-session basis, as well as across days. Thus, PFC beta is likely correlated with rule-learning, which increases across sessions and days.

Minor Comments 1) The manuscript reports that within-day normalization eliminates the negative correlation between PFC beta power and CA1 SWR properties, which is an interesting observation. Because animals performed four sessions per day, it would be informative to examine whether PFC beta power itself changes across the four sessions within a day over the 5-day period, in addition to the day- averaged beta in Fig. 2.

We agree and the per session analysis in Fig. 5 address this issue.

Similarly, CA1 SWR properties could be analyzed at the session level across days Fig. 3. Such analyses would provide readers with a clearer sense of the timescale over which PFC beta and CA1 SWRs evolve, and would help contextualize the experience-dependent dynamics reported in the study.

This is an additional analysis we performed to address this important point. We performed session-by-session analysis for SWR rate and size, and did not find a within day correlation (Fig. 5). This suggests the SWR changes are related to changes in the familiarity across days rather than performance changes within day. This contrasts with the within-day correlation between PFC beta power and performance (response to comment 7 above).

2) Line 230: The phrase "Given that prior studies..." should include appropriate citations.

We have added the missing citations.

3) In Figs. 1A and 2A, it is unclear what the second vertical dashed line after goal entry represents. The legend should clarify whether this indicates reward delivery, the end of the 850 ms window, or another event.

We have clarified the dotted line is marks 1 s after goal entry. The time scale is more clearly shown in these figures as well.

4) Fig. S1B (right): The y-axis label is cut off.

We corrected this error.

5) The unit of "power" (e.g., µV²) should be indicated on the y-axis in Figs. 5 and S3.

We have added the missing units. The previous Fig. S3 is now Fig. 6-1.

6) "SWR size" is not defined and should be described in the Methods.

We have clarified the definition of SWR size in the methods. "The SWR size was calculated as the mean standard deviation above the mean ripple frequency band filtered signal for the duration of the SWR." 7) SWR rate was calculated over the first 10 seconds after goal entry, excluding shorter visits. The manuscript should report the average reward consumption duration and justify this threshold.

We clarified why we chose 10s and this was based on the movement pattern at the goal. Here are the speed profiles for the animal relative to goal entry and 10s is when animal can start to leave the goal (Fig. 2-4).

8) A fixed 500 ms window (350-850 ms after nose poke) was used to quantify beta power. A more quantitative approach would detect beta onset and offset per session, especially since beta peak timing appears to shift over days (Fig. S1).

Based on this useful suggestion, we have changed how we define the window for quantifying the power. We now use a 500ms window centered on the peak of each day's beta power for each animal. This captures the variation between days and animals. The main findings remain unchanged.

## References

[B1] Allen TA, Salz DM, McKenzie S, Fortin NJ (2016) Nonspatial sequence coding in CA1 neurons. J Neurosci 36:1547–1563. 10.1523/JNEUROSCI.2874-15.2016 PMC4737769 26843637

[B2] Ambrose RE, Pfeiffer BE, Foster DJ (2016) Reverse replay of hippocampal place cells is uniquely modulated by changing reward. Neuron 91:1124–1136. 10.1016/j.neuron.2016.07.047 PMC6013068 27568518

[B3] Buzsáki G (1989) Two-stage model of memory trace formation: a role for “noisy” brain states. Neuroscience 31:551–570. 10.1016/0306-4522(89)90423-5 2687720

[B4] Buzsáki G (1996) The hippocampo-neocortical dialogue. Cereb Cortex 6:81–92. 10.1093/cercor/6.2.81 8670641

[B5] Buzsáki G (2015) Hippocampal sharp wave-ripple: a cognitive biomarker for episodic memory and planning. Hippocampus 25:1073–1188. 10.1002/hipo.22488 PMC4648295 26135716

[B6] Carey AA, Tanaka Y, van der Meer MAA (2019) Reward revaluation biases hippocampal replay content away from the preferred outcome. Nat Neurosci 22:1450–1459. 10.1038/s41593-019-0464-6 31427771

[B7] Cheng S, Frank LM (2008) New experiences enhance coordinated neural activity in the hippocampus. Neuron 57:303–313. 10.1016/j.neuron.2007.11.035 PMC2244590 18215626

[B8] Chernyshev BV, Pultsina KI, Tretyakova VD, Miasnikova AS, Prokofyev AO, Kozunova GL, Stroganova TA (2023) Losses resulting from deliberate exploration trigger beta oscillations in frontal cortex. Front Neurosci 17:1152926. 10.3389/fnins.2023.1152926 PMC10211346 37250414

[B9] Chu JP, Kemere CT (2021) GhostiPy: an efficient signal processing and spectral analysis toolbox for large data. eNeuro 8:ENEURO.0202-21.2021. 10.1523/ENEURO.0202-21.2021 PMC8641918 34556557

[B10] Cohen MX, Elger CE, Ranganath C (2007) Reward expectation modulates feedback-related negativity and EEG spectra. Neuroimage 35:968–978. 10.1016/j.neuroimage.2006.11.056 PMC1868547 17257860

[B11] Cohen MX, Wilmes K, Vijver I (2011) Cortical electrophysiological network dynamics of feedback learning. Trends Cogn Sci 15:558–566. 10.1016/j.tics.2011.10.004 22078930

[B12] Cohen Y, Putrino D, Wilson DA (2015) Dynamic cortical lateralization during olfactory discrimination learning. J Physiol 593:1701–1714. 10.1113/jphysiol.2014.288381 PMC4386967 25604039

[B13] Cunningham PJ, Redish AD (2025) Opposing, multiplexed information in lateral and ventral orbitofrontal cortex guides sequential foraging decisions in rats. Neuron 113:3031–3047.e3036. 10.1016/j.neuron.2025.06.009 PMC12313183 40695283

[B14] Dalton GL, Wang NY, Phillips AG, Floresco SB (2016) Multifaceted contributions by different regions of the orbitofrontal and medial prefrontal cortex to probabilistic reversal learning. J Neurosci 36:1996–2006. 10.1523/JNEUROSCI.3366-15.2016 PMC6602019 26865622

[B15] Diehl GW, Redish AD (2023) Differential processing of decision information in subregions of rodent medial prefrontal cortex. Elife 12:e82833. 10.7554/eLife.82833 PMC9848391 36652289

[B16] Engel AK, Fries P (2010) Beta-band oscillations–signalling the status quo? Curr Opin Neurobiol 20:156–165. 10.1016/j.conb.2010.02.015 20359884

[B17] Foster DJ, Wilson MA (2006) Reverse replay of behavioural sequences in hippocampal place cells during the awake state. Nature 440:680–683. 10.1038/nature04587 16474382

[B18] Fourcaud-Trocmé N, Lefèvre L, Garcia S, Messaoudi B, Buonviso N (2019) High beta rhythm amplitude in olfactory learning signs a well-consolidated and non-flexible behavioral state. Sci Rep 9:20259. 10.1038/s41598-019-56340-y PMC6937317 31889074

[B19] Frank LM, Brown EN, Wilson M (2000) Trajectory encoding in the hippocampus and entorhinal cortex. Neuron 27:169–178. 10.1016/S0896-6273(00)00018-0 10939340

[B20] Frederick DE, Brown A, Brim E, Mehta N, Vujovic M, Kay LM (2016) Gamma and beta oscillations define a sequence of neurocognitive modes present in odor processing. J Neurosci 36:7750–7767. 10.1523/JNEUROSCI.0569-16.2016 PMC4951578 27445151

[B21] Gervais R, Buonviso N, Martin C, Ravel N (2007) What do electrophysiological studies tell us about processing at the olfactory bulb level? J Physiol Paris 101:40–45. 10.1016/j.jphysparis.2007.10.006 18054211

[B22] Golebiowska J, Rygula R (2017) Lesions of the orbitofrontal but not medial prefrontal cortex affect cognitive judgment bias in rats. Front Behav Neurosci 11:51. 10.3389/fnbeh.2017.00051 PMC5359275 28377703

[B24] HajiHosseini A, Holroyd CB (2015) Reward feedback stimuli elicit high-beta EEG oscillations in human dorsolateral prefrontal cortex. Sci Rep 5:13021. 10.1038/srep13021 PMC4538377 26278335

[B23] HajiHosseini A, Hutcherson CA, Holroyd CB (2020) Beta oscillations following performance feedback predict subsequent recall of task-relevant information. Sci Rep 10:15114. 10.1038/s41598-020-72128-x PMC7493892 32934274

[B25] Hervig ME, Fiddian L, Piilgaard L, Božič T, Blanco-Pozo M, Knudsen C, Robbins TW (2020) Dissociable and paradoxical roles of rat medial and lateral orbitofrontal cortex in visual serial reversal learning. Cereb Cortex 30:1016–1029. 10.1093/cercor/bhz144 PMC7132932 31343680

[B26] Hong DD, Huang WQ, Ji AA, Yang SS, Xu H, Sun KY, Yu P (2019) Neurons in rat orbitofrontal cortex and medial prefrontal cortex exhibit distinct responses in reward and strategy-update in a risk-based decision-making task. Metab Brain Dis 34:417–429. 10.1007/s11011-018-0360-x 30535618

[B27] Hoy CW, de Hemptinne C, Wang SS, Harmer CJ, Apps MAJ, Husain M, Little S (2024) Beta and theta oscillations track effort and previous reward in the human basal ganglia and prefrontal cortex during decision making. Proc Natl Acad Sci U S A 121:e2322869121. 10.1073/pnas.2322869121 PMC11295073 39047043

[B28] Huelin Gorriz M, Takigawa M, Bendor D (2023) The role of experience in prioritizing hippocampal replay. Nat Commun 14:8157. 10.1038/s41467-023-43939-z PMC10710481 38071221

[B29] Igarashi KM, Lu L, Colgin LL, Moser MB, Moser EI (2014) Coordination of entorhinal-hippocampal ensemble activity during associative learning. Nature 510:143–147. 10.1038/nature13162 24739966

[B30] Iturra-Mena AM, Kangas BD, Luc OT, Potter D, Pizzagalli DA (2023) Electrophysiological signatures of reward learning in the rodent touchscreen-based probabilistic reward task. Neuropsychopharmacology 48:700–709. 10.1038/s41386-023-01532-4 PMC9938210 36646816

[B31] Jadhav SP, Kemere C, German PW, Frank LM (2012) Awake hippocampal sharp-wave ripples support spatial memory. Science 336:1454–1458. 10.1126/science.1217230 PMC4441285 22555434

[B32] Jayachandran M, Viena TD, Garcia A, Veliz AV, Leyva S, Roldan V, Allen TA (2023) Nucleus reuniens transiently synchronizes memory networks at beta frequencies. Nat Commun 14:4326. 10.1038/s41467-023-40044-z PMC10356781 37468487

[B33] Kawasaki M, Yamaguchi Y (2013) Frontal theta and beta synchronizations for monetary reward increase visual working memory capacity. Soc Cogn Affect Neurosci 8:523–530. 10.1093/scan/nss027 PMC3682435 22349800

[B34] Kay K, Sosa M, Chung JE, Karlsson MP, Larkin MC, Frank LM (2016) A hippocampal network for spatial coding during immobility and sleep. Nature 531:185–190. 10.1038/nature17144 PMC5037107 26934224

[B35] Kay LM, Lancaster LR, Freeman WJ (1996) Reafference and attractors in the olfactory system during odor recognition. Int J Neural Syst 7:489–495. 10.1142/s0129065796000476 8968840

[B36] Kay LM, Freeman WJ (1998) Bidirectional processing in the olfactory-limbic axis during olfactory behavior. Behav Neurosci 112:541–553. 10.1037//0735-7044.112.3.541 9676972

[B37] Kim SM, Frank LM (2009) Hippocampal lesions impair rapid learning of a continuous spatial alternation task. PLoS One 4:e5494. 10.1371/journal.pone.0005494 PMC2674562 19424438

[B38] Koloski MF, Hulyalkar S, Barnes SA, Mishra J, Ramanathan DS (2024a) Cortico-striatal beta oscillations as a reward-related signal. Cogn Affect Behav Neurosci 24:839–859. 10.3758/s13415-024-01208-6 PMC11390840 39147929

[B39] Koloski MF, O'Hearn CM, Frankot M, Giesler LP, Ramanathan DS, Vonder Haar C (2024b) Behavioral interventions can improve brain injury-induced deficits in behavioral flexibility and impulsivity linked to impaired reward-feedback beta oscillations. J Neurotrauma 41:e1721–e1737. 10.1089/neu.2023.0448 PMC11339556 38450560

[B40] Koloski MF, Salimi M, Hulyalkar S, Tang T, Barnes SA, Mishra J, Ramanathan DS (2025) Beta and high gamma oscillations in the cortico-striatal network reflect reward certainty on a probabilistic reversal learning task. J Neurosci 45:e0858252025. 10.1523/JNEUROSCI.0858-25.2025 PMC12491767 40858499

[B41] Kumaran D, Hassabis D, McClelland JL (2016) What learning systems do intelligent agents need? Complementary learning systems theory updated. Trends Cogn Sci 20:512–534. 10.1016/j.tics.2016.05.004 27315762

[B42] Larkin MC, Lykken C, Tye LD, Wickelgren JG, Frank LM (2014) Hippocampal output area CA1 broadcasts a generalized novelty signal during an object-place recognition task. Hippocampus 24:773–783. 10.1002/hipo.22268 PMC4065199 24596296

[B43] Lundqvist M, Miller EK, Nordmark J, Liljefors J, Herman P (2024) Beta: bursts of cognition. Trends Cogn Sci 28:662–676. 10.1016/j.tics.2024.03.010 38658218

[B44] Maharjan DM, Dai YY, Glantz EH, Jadhav SP (2018) Disruption of dorsal hippocampal - prefrontal interactions using chemogenetic inactivation impairs spatial learning. Neurobiol Learn Mem 155:351–360. 10.1016/j.nlm.2018.08.023 PMC6365178 30179661

[B45] Marco-Pallarés J, Münte TF, Rodríguez-Fornells A (2015) The role of high-frequency oscillatory activity in reward processing and learning. Neurosci Biobehav Rev 49:1–7. 10.1016/j.neubiorev.2014.11.014 25464028

[B46] Martin C, Gervais R, Hugues E, Messaoudi B, Ravel N (2004) Learning modulation of odor-induced oscillatory responses in the rat olfactory bulb: a correlate of odor recognition? J Neurosci 24:389–397. 10.1523/JNEUROSCI.3433-03.2004 PMC6730004 14724237

[B47] Martin C, Beshel J, Kay LM (2007) An olfacto-hippocampal network is dynamically involved in odor-discrimination learning. J Neurophysiol 98:2196–2205. 10.1152/jn.00524.2007 17699692

[B48] McClelland JL, McNaughton BL, Oreilly RC (1995) Why there are complementary learning-systems in the hippocampus and neocortex - insights from the successes and failures of connectionist models of learning and memory. Psychol Rev 102:419–457. 10.1037/0033-295x.102.3.419 7624455

[B49] Miles JT, Kidder KS, Mizumori SJY (2023) Hippocampal beta rhythms as a bridge between sensory learning and memory-guided decision-making. Front Syst Neurosci 17:1187272. 10.3389/fnsys.2023.1187272 PMC10196064 37215359

[B50] Nakagawa S, Schielzeth H (2013) A general and simple method for obtaining R2 from generalized linear mixed-effects models. Methods Ecol Evol 4:133–142. 10.1111/j.2041-210x.2012.00261.x

[B51] Paxinos G, Watson C (2007) *The rat brain in stereotaxic coordinates*, Ed 6. San Diego: Academic Press.

[B52] Shin EJ, Jang Y, Kim S, Kim H, Cai X, Lee H, Jung MW (2021) Robust and distributed neural representation of action values. Elife 10:e53045. 10.7554/eLife.53045 PMC8104958 33876728

[B54] Singer AC, Frank LM (2009) Rewarded outcomes enhance reactivation of experience in the hippocampus. Neuron 64:910–921. 10.1016/j.neuron.2009.11.016 PMC2807414 20064396

[B53] Singer AC, Carr MF, Karlsson MP, Frank LM (2013) Hippocampal SWR activity predicts correct decisions during the initial learning of an alternation task. Neuron 77:1163–1173. 10.1016/j.neuron.2013.01.027 PMC3751175 23522050

[B55] Spitzer B, Haegens S (2017) Beyond the status quo: a role for beta oscillations in endogenous content (Re)Activation. eNeuro 4:ENEURO.0170-17.2017. 10.1523/ENEURO.0170-17.2017 PMC5539431 28785729

[B56] Sul JH, Kim H, Huh N, Lee D, Jung MW (2010) Distinct roles of rodent orbitofrontal and medial prefrontal cortex in decision making. Neuron 66:449–460. 10.1016/j.neuron.2010.03.033 PMC2872629 20471357

[B57] Symanski CA, Bladon JH, Kullberg ET, Miller P, Jadhav SP (2022) Rhythmic coordination and ensemble dynamics in the hippocampal-prefrontal network during odor-place associative memory and decision making. Elife 11:79545. 10.7554/eLife.79545 PMC9799972 36480255

[B58] Tang W, Shin JD, Frank LM, Jadhav SP (2017) Hippocampal-prefrontal reactivation during learning is stronger in awake compared with sleep states. J Neurosci 37:11789–11805. 10.1523/JNEUROSCI.2291-17.2017 PMC5719968 29089440

[B59] van der Meer MAA, Bendor D (2025) Awake replay: off the clock but on the job. Trends Neurosci 48:257–267. 10.1016/j.tins.2025.02.006 40121166

[B60] Verharen JPH, den Ouden HEM, Adan RAH, Vanderschuren LJMJ (2020) Modulation of value-based decision making behavior by subregions of the rat prefrontal cortex. Psychopharmacology 237:1267–1280. 10.1007/s00213-020-05454-7 PMC7196947 32025777

[B61] Voigts J, Newman JP, Wilson MA, Harnett MT (2020) An easy-to-assemble, robust, and lightweight drive implant for chronic tetrode recordings in freely moving animals. J Neural Eng 17:026044. 10.1088/1741-2552/ab77f9 PMC8878001 32074511

[B62] Voloh B, Oemisch M, Womelsdorf T (2020) Phase of firing coding of learning variables across the fronto-striatal network during feature-based learning. Nat Commun 11:4669. 10.1038/s41467-020-18435-3 PMC7495418 32938940

[B63] Wang G, Zhou N, Leveroni ZM, Toptas P, Kaye AE, Gutierrez J, Rodriguez-Leon A, Yu JY (2026) Distinct goal location beta frequency dynamics in hippocampus and prefrontal cortex across learning. bioRxiv 2025.08.11:669642. 10.1101/2025.08.11.669642

[B64] Womelsdorf T, Valiante TA, Sahin NT, Miller KJ, Tiesinga P (2014) Dynamic circuit motifs underlying rhythmic gain control, gating and integration. Nat Neurosci 17:1031–1039. 10.1038/nn.3764 25065440

[B65] Xiao J, Adkinson JA, Myers J, Allawala AB, Mathura RK, Pirtle V, Sheth SA (2024) Beta activity in human anterior cingulate cortex mediates reward biases. Nat Commun 15:5528. 10.1038/s41467-024-49600-7 PMC11250824 39009561

